# Synthesis of Novel Benzodifuranyl; 1,3,5-Triazines; 1,3,5-Oxadiazepines; and Thiazolopyrimidines Derived from Visnaginone and Khellinone as Anti-Inflammatory and Analgesic Agents

**DOI:** 10.3390/molecules25010220

**Published:** 2020-01-05

**Authors:** Ameen Ali Abu-Hashem, Sami A Al-Hussain, Magdi E. A. Zaki

**Affiliations:** 1 Photochemistry Department, Heterocyclic Unit, National Research Centre, Dokki, Giza12622, Egypt; meazaki@gmail.com; 2 Chemistry Department, Faculty of Science, Jazan University, 45142 Jazan, Saudi Arabia; 3 Department of Chemistry, Faculty of Science, Al-Imam Mohammad Ibn Saud Islamic University (IMSIU), Riyadh 13318, Saudi Arabia; sahussain@imamu.edu.sa

**Keywords:** visnagenone, khellinone, benzodifuran, pyrimidine, piperazine, morpholine, thiazole, triazine, oxadiazepine, anti-inflammatory, analgesic activity

## Abstract

Novel (4-methoxy or 4,8-dimethoxy)-3-methyl-*N*-(6-oxo-2-thioxo-1,2,3, 6-tetrahydro- pyrimidin-4-yl) benzo [1,2-b: 5, 4-b’] difuran-2-carboxamide (**5a**–**b**) has been synthesized by the reaction of visnagenone–ethylacetate (**2a**) or khellinone–ethylacetate (**2b**) with 6-aminothiouracil in dimethylformamide or refluxing of benzofuran-oxy-*N*-(2-thioxopyrimidine) acetamide (**4a**–**b**) in sodium ethoxide to give the same products (**5a**,**b**) in good yields. Thus, compounds **5a**–**b** are used as an initiative to prepare many new heterocyclic compounds such as 2-(4-(3-methylbenzodifuran- 2-carbox-amido) pyrimidine) acetic acid (**6a**–**b**), *N*-(thiazolo[3, 2-a]pyrimidine)-3-methylbenzo- difuran-2-carboxamide (**7a**–**b**), *N*-(2-thioxopyrimidine)-methylbenzodifuran-2-carbimidoylchloride (8a–b), N-(2-(methyl-thio) pyrimidine)-3-methylbenzodifuran-2-carbimidoylchloride (9a–b), N-(2, 6 -di(piperazine or morpholine)pyrimidine)-1-(3-methylbenzodifuran)-1-(piperazine or morpholine) methanimine(10a–d), 8-(methylbenzodifuran)-thiazolopyrimido[1,6-a][1,3,5]triazine-3,5-dione (11a –b), 8-(3-methyl benzodifuran)-thiazolopyrimido[6,1-d][1,3,5]oxadiazepine-trione (12a–b), and 2,10 -di(sub-benzylidene)-8-(3-methylbenzodifuran)-thiazolopyrimido[6,1-d][1,3,5]oxadiazepine-3,5,11- trione (13a–f). All new chemical structures were illustrated on the basis of elemental and spectral analysis (IR, NMR, and MS). The new compounds were screened as cyclooxygenase-1/ cyclooxygenase-2 (COX-1/COX-2) inhibitors and had analgesic and anti-inflammatory activities. The compounds **10a**–**d** and **13a**–**f** had the highest inhibitory activity on COX-2 selectivity, with indices of 99–90, analgesic activity of 51–42% protection, and anti-inflammatory activity of 68%–59%. The inhibition of edema for the same compounds, **10a**–**d** and **13a**–**f**, was compared with sodium diclofenac as a standard drug.

## 1. Introduction

**Figure 1:**
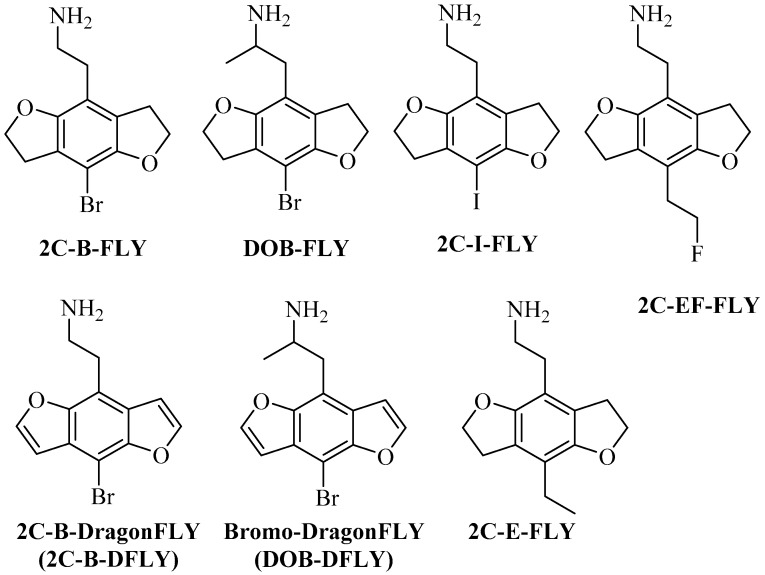
Chemical structures of the phenyl-alkyl-amine hallucinogens 2,5-dimethoxy-4-bromo- phenethylamine (2C-B) and 2,5-dimethoxy-4-bromoamphetamine (DOB), 2-(8-iodo-2,3,6,7-tetra hydrobenzo[1,2-b:4,5-b`]difuran-4-yl) ethan-1-amine (2C-I-FLY), 2-(8-ethyl-2,3,6,7-tetrahydrobenzo [1,2-b:4,5-b`]difuran-4-yl)ethan-1-amine (2C-E-FLY), 2-[8-2-fluoroethyl)-2,3,6,7-tetrahydrobenzo[1,2- b:4,5-b`]difuran-4-yl]ethan-1-amine (2C-EF-FLY), also their benzodifuran and tetrahydrobenzodi- furan analogues.

Visnagin, khellin, visnaginone, and khellinone have been extracted from *Ammi visnaga Lam* and other plants. These compounds belong to a family of natural furochromones. Previous and recent scientific research has shown that furochromones have a wide range of biological activities [1] in the treatment of many diseases, including antimicrobial [2], anticancer [3], anti-inflammatory, and analgesic activities [4], the ability to bind to DNA [5], the treatment of vitiligo and psoriasis [6], the treatment of spasms and kidney stones [7], and the treatment of urolithiasis and hypertriglyceridemia [8,9]. Benzofurans and furochromones are used to treat viral and cancer cell activities [10,11]. In addition, several heterocyclic moieties such as thiazolidinones [12], chalcones [13], sulfonamides [14], isoxazole [15], and Mannich bases [16], when linked with benzofuran (visnagenone, khellinone) derivatives, offer several biological activities. Benzofuran derivatives, such as machicendiol, which is extracted from Machilus glaucescens natural plants, are medicinal compounds that have been used in many treatments for ulcers, asthma, and rheumatism [17]. Ailanthoidol, a neolignan derivative, has antiviral, antioxidant, and antifungal activities [18]. In addition, benzofuran, when fused with benzocarbazoles, shows potential antitumor and antibiotic activities [19]. Likewise, substituted benzofurans are active in promoting insulin sensitivity [20]. Furthermore, benzofurans containing 1, 3-thiazole derivatives possess tuberculostatic, antifungal, and antibacterial activities [21,22,23]. In recent years, substances analogous to phenyl-alkyl-amine hallucinogens have appeared as recreational drugs, including 2,5-dimethoxy-4-bromophenethyl- amine (2C-B), 2,5-dimethoxy-4-bromoamphetamine (DOB), (2-(8-bromo-2,3,6,7-tetrahydrobenzo[1,2-b:4,5-b`]difuran-4-yl)ethan-1-amine (2C-B-FLY), (1-(8-bromo-2,3,6,7-tetrahydrobenzo[1,2-b:4,5-b`] difuran-4-yl)propan-2-amine (DOB-FLY), (2-(8-bromobenzo[1,2-b:4,5-b`]difuran-4-yl)ethan-1-amine (2C-B-DFLY), (1-(8-bromo benzo[1,2-b:4,5-b`]difuran-4-yl)propan-2-amine (DOB-DFLY), 2-(8-iodo-2,3,6,7-tetrahydrobenzo [1,2-b:4,5-b`]difuran-4-yl) ethan-1-amine (2C-I-FLY), 2-(8-ethyl-2,3,6,7-tetra- hydrobenzo[1,2-b:4,5-b`]difuran-4-yl)ethan-1-amine(2C-E-FLY) and 2-[8-2-fluoroethyl)-2,3,6,7-tetra-hydrobenzo[1,2-b: 4,5-b`] difuran-4-yl]ethan-1-amine (2C-EF-FLY) [24,25], as shown in Figure 1.

The enzyme cyclooxygenase (COX) secretes prostaglandins within the cell. There are two types of COX enzymes: cycloxygenase-1 (COX-1) and cycloxygenase-2 (COX-2). Inside the body, different roles of the enzyme cyclooxygenase (COX) produce prostaglandins (PGs), which is the main pathway is COX-2 that promotes pain, inflammation, and fever. Moreover, COX-1 generates prostaglandins that activate platelets and safeguard the stomach and intestinal lining. Older NSAIDs block COX-1 and COX-2, but new COX-2 inhibitors only block the COX-2 enzyme. COX-2 inhibitors do not block COX-1, and they do not bring about ulcers or increase the danger of bleeding as much as the older NSAIDs have. Sodium diclofenac is an older NSAID, and it decreases the production of prostaglandin via blocking both cycloxygenase-1 (COX-1) and cycloxygenase-2 (COX-2) [26]. In the early 1990s, a new isoform of COX was discovered in the development of anti-inflammatory agents and had the best safety profile in contrast to older NSAIDs [3,4]. The drug celecoxib is a selective COX-2 inhibitor [27]. Novel drugs have been discovered through synthesizing and developing new heterocyclic compounds, including NSAIDs and selective COX-2 inhibitors [28]. This article is an extension of our previous work on the synthesis of heterocyclic compounds resulting from natural furochromones (visnagin and khellin), which have biological activity [1,2,3,4,5,22,23,29,30,31,32,33,34]. In the current article, compounds benzodifuran; 1,3,5-triazine; 1,3,5-oxadiazepine; and thiazolopyrimidine derivatives were synthesized from furochromones and evaluated as COX1/COX-2 inhibitors as well as analgesic and anti-inflammatory agents.

## 2. Results and Discussion

### 2.1. Synthesis

**Scheme 1:**
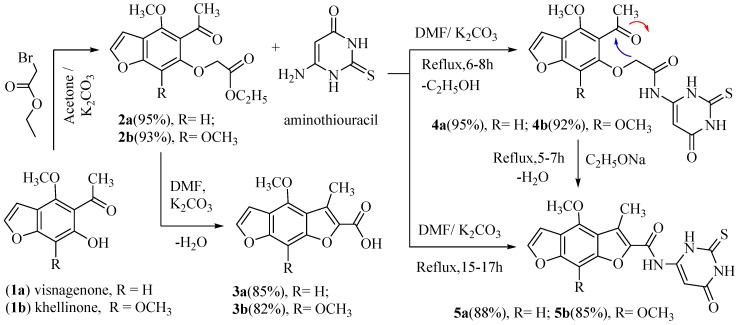
Synthesis of *N*-(6-oxo-2-thioxo-1,2,3,6-tetrahydropyrimidin-4-yl) benzo [1,2-b: 5,4-b`] difuran-2-carboxamide.

Hydrolysis of the naturalistic furochromones (visnagin and khellin) via potassium hydroxide gave visnaginone (**1a**) and khellinone (**1b**). The same compounds (**1a** and **1b**) reacted with ethyl bromoacetate in the presence of potassium carbonate yielded (4-methoxy or 4, 7-dimethoxy)-5-acetyl benzofuran-6-yloxy) acetic acid ethyl ester (**2a** and **2b**), respectively. The condensation of (**2a)** or **(2b)** in dimethylformamide and anhydrous potassium carbonate with refluxing for four hours produced (4-methoxy or 4, 8-dimethoxy)-3-methylbenzo [1,2-b: 5,4-b`] difuran-2-carboxylic acid (**3a** and **3b**) [5]. In this manuscript, the refluxing of (**2a** or **2b**) with 6-aminothiouracil in DMF and anhydrous potassium carbonate [2] for 6–8 h (a short time) or 15–17 h (a long time) under control thin layer chromatography afforded new compounds 2-((5-acetyl-(4-methoxy or 4,7-dimethoxy)-benzofuran-6-yl) oxy)-*N*-(6-oxo-2- thioxo-1,2,3,6-tetrahydropyrimidin-4-yl) acetamide (**4a**,**b**) and (4-methoxy or 4, 8-dimethoxy)-3-methyl-*N*-(6-oxo-2-thioxo-1,2,3,6-tetra-hydropyrimidin-4-yl) benzo[1,2-b: 5,4-b`]difuran-2-carboxamide (**5a, b**), respectively, in good yield. As well, in another way, heating **4a** or **4b** under reflux in sodium ethoxide solution for 5–7 h awarded similar products **5a** and **5b**, respectively. The ^1^H-NMR spectrum of compound **4a** displayed one singlet signal at 4.70 ppm corresponding to the two protons of the CH_2_ and three singlet broad signals at 8.50, 9.70, and 11.90 ppm corresponding to the three protons of the three NH groups (D_2_O exchangeable). The ^1^H-NMR spectrum of compound **5a** exposed three singlet broad signals at 8.60, 9.80, and 12.10 ppm, conforming to the three protons of the three NH groups (D_2_O exchangeable). The mass spectra of **4a**, **4b**, **5a,** and **5b** indicated molecular ion peaks at *m*/*z* 389 (M^+^, 100%), 419 (M^+^, 100%), 371 (M^+^, 100%), and 401 (M+, 100%), respectively (Scheme 1).

**Scheme 2:**
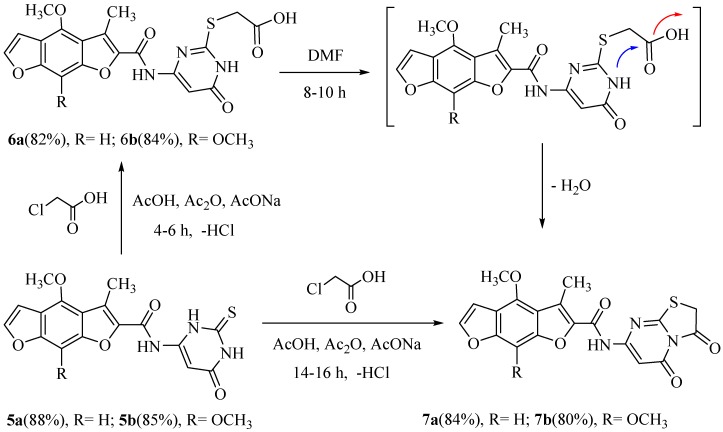
Synthesis of *N*-(3,5-dioxo-2, 3-dihydro-5*H*-thiazolo [3, 2-a] pyrimidin-7-yl)-3-methyl benzo [1,2-b: 5,4-b`] difuran-2-carboxamide.

Moreover, the reaction of **5a** or **5b** with chloroacetic acid in a mixture of glacial acetic acid/acetic anhydride and anhydrous sodium acetate for 4–6 h (a short time) or 14–16 h (a long time) under control (TLC) afford the new compounds 2-((4-((4-methoxy or 4, 8-dimethoxy)-3-methyl benzo[1,2-b: 5,4-b`]difuran-2-carboxamido)-6-oxo-1,6-dihydropyrimidin-2-yl) thio) acetic acid (**6a**, **b**) and *N*-(3,5- dioxo-2,3-dihydro-5*H*-thiazolo[3,2-a]pyrimidin-7-yl)-(4-methoxy or 4,8-dimethoxy)-3-methyl benzo [1,2-b: 5,4- b`]difuran-2-carboxamide (**7a,b**) in good yields. In addition, the refluxing of **6a** or **6b** in dimethylformamide solution produced the same products, **7a** and **7b**. The IR spectrum of compound **6a** showed the presence of a broad band absorption at 3380–3382 cm^−1^ of two NH groups, 3350 cm^−1^ of one OH group, 1753 cm^−1^ of the acid carbonyl group, and 1687 and 1681 cm^−1^ of the two amide carbonyl groups. The ^1^H-NMR spectrum of **6a** exhibited three singlet broad signals at 10.10, 11.95, and 12.50 ppm conforming to the three protons of the two NH and one OH groups, respectively (with D_2_O exchangeable). Similarly, the ^1^H-NMR spectrum of **7a** demonstrated five singlet signals at *δ* 2.38, 4.01, 4.20, 6.95, and 7.05 ppm, approving the 10 protons of the CH_3_, OCH_3_, CH_2_, CH-pyrimidine, and CH-phenyl groups, respectively, and one singlet broad signal at 10.20 ppm corresponding to the one proton of the one NH group (D_2_O exchangeable). The chemical structures of new compounds were assured by spectral data (IR, ^1^H, ^13^C-NMR, MS) and elemental analysis, as shown in the experimental section (Scheme 2). 

**Scheme 3:**
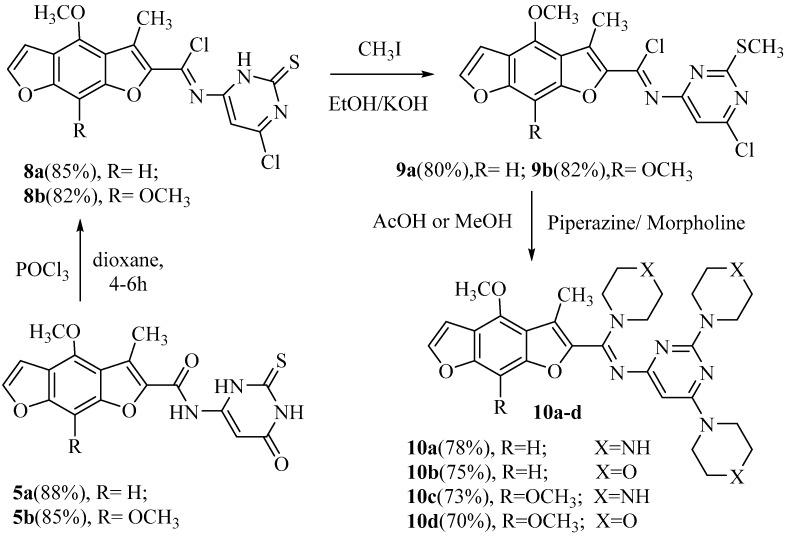
Synthesis of *N*-(2, 6-di (piperazin-1-yl or morpholino) pyrimidinyl)-1-((4-methoxy or 4, 8- dimethoxy)-3-methylbenzo [1,2-b: 5,4-b`] difuranyl)-1-(piperazin-1-yl or morpholino) methanimine.

Furthermore, the reaction of **5a**, **b** with phosphorus oxychloride in dry dioxane [4] yielded *N*-(6-chloro-2-thioxo-2, 3-dihydropyrimidin-4-yl)-(4-methoxy or 4, 8-dimethoxy)-3-methyl benzo [1,2-b: 5,4-b`] difuran-2-carbimidoyl chloride **(8a**, **b)**. The IR spectra of **8a**, **b** exposed the absence of some absorption bands in the two NH and two carbonyl groups. The alkylation of an ethanolic potassium hydroxide solution of **8a** or **8b** with methyl iodide afforded *N*-(6-chloro-2-(methylthio) pyrimidin-4-yl)-(4-methoxy or 4, 8-dimethoxy)-3-methylbenzo [1,2-b: 5,4-b`] difuran-2-carbimidoyl chloride **(9a, b)** in good yield. The ^1^H-NMR spectra of **9a** and **9b** displayed singlets at *δ* 2.25 and 2.23 ppm, respectively, agreeing to a SCH_3_ group. The latter compounds, **9a**,**b**, having an active chlorine substituent, reacted with each piperazine or morpholine in boiling methanol or glacial acetic acid to produce *N*-(2, 6-di (piperazin-1-yl or morpholino) pyrimidin-4-yl)-1-((4-methoxy or 4, 8- dimethoxy )-3-methylbenzo [1,2-b: 5,4-b`] difuran-2-yl)-1-(piperazin-1-yl or morpholino) methanimine **(10a–d).** The IR spectra of **10a** and **10c** revealed strong absorption broad bands at 3385–3390 cm^–1^ characteristic of the (br, NH) groups. The ^1^H-NMR spectrum of **10a** indicated three singlet broad signals at *δ* 10.10, 10.30, and 10.50 ppm conforming to the three protons of the three NH groups, which were D_2_O exchangeable. All new compounds were confirmed by their correct compatible spectrum data and elemental analyses values (see the Experimental section, Scheme 3).

**Scheme 4:**
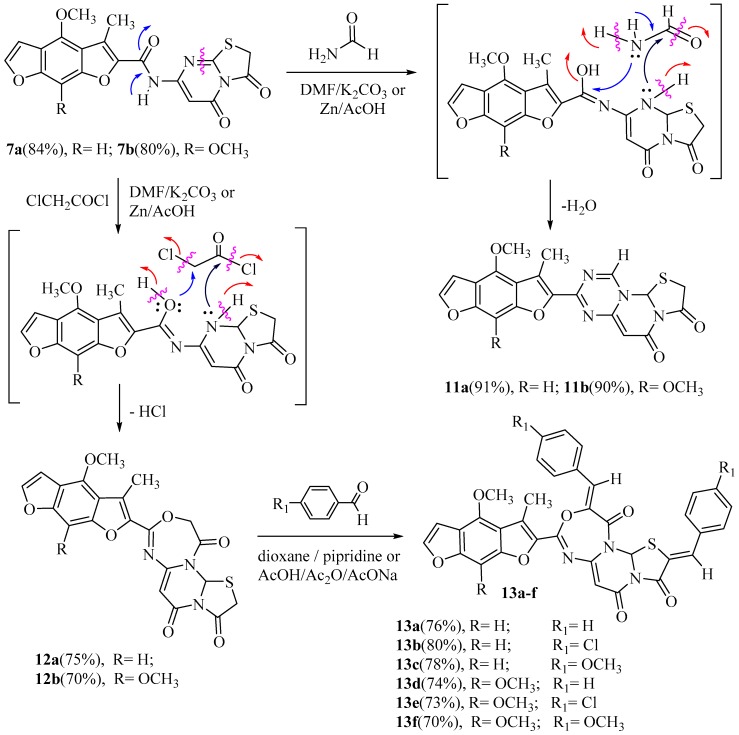
Synthesis of 1,3,5-triazine and 1,3,5-oxadiazepine and substituted-benzylidene-3- methylbenzo [1,2-b:5,4-b`]difuran-2-yl)-5*H*,12a*H*-thiazolo[2’,3’:2,3]pyrimidine derivatives.

Similarly, in the first method, compound **7a** or **7b** reacted with formamide or α-halo-ketones as chloroacetyl-chloride in DMF, in the presence of anhydrous potassium carbonate to afford the corresponding 8-(4-methoxy or 4,8-dimethoxy-3-methylbenzo[1,2-b:5,4-b`]difuran-2-yl)-2-hydro-5H, 11aH-thiazolo[2’,3’:2,3]pyrimido[1,6-a][1,3,5]triazine-3,5-dione (11a,b), and 8-(4-methoxy or 4,8- dimethoxy-3-methylbenzo[1,2-b:5,4-b`]difuran-2-yl)-5H,12aH-thiazolo[2’,3’:2,3]pyrimido[6,1-d][1,3,5]oxadiazepine-3,5,11(2H,10H)-trione (12a,b) respectively, in good yields. In addition, the second method involved the refluxing of 7a or 7b with formamide or chloroacetyl-chloride in acetic acid, in the presence of zinc dust, to afford the conforming the same products (11a–b and 12a–b), respectively. The formation of 11a–b and 12a–b from the corresponding 7a–b may proceed through an initial reduction of compounds 7a–b, followed by the cyclocondensation of the intermediates produced with the ketones followed by a necessary final reduction step to produce 11a, b and 12a, b. The IR spectrum of 11a showed absorption bands at 1684 and 1680 cm–1 of two carbonyl amide groups and 1632 cm–1 to the C=N group. The 1H-NMR spectrum of 11a revealed six singlet signals at δ 2.32, 3.94, 5.85, 7.20, 7.35, and 8.10 ppm, agreeing with the three protons of methyl, three protons of methoxy, one proton of thiazole, one proton of phenyl, one proton of triazine, and one proton of the pyrimidine ring, respectively and exhibited two doublet signals at δ 4.10 and 4.17 ppm approving the two protons of CH2, thiazole ring. Hence, CH2 protons are diastereotopic pairs to give the germinal coupling (J = 6.90 Hz). The 13 C-NMR spectrum of 11a exhibited signals at δ 166.7 and 169.8 ppm corresponding to two carbon atoms of the two carbonyl groups. Likewise, the IR spectrum of 12a displayed absorption bands at 1688, 1684, and 1679 cm–1 corresponding to three carbonyl amide groups, respectively. Besides, the 13C-NMR spectrum of 12a showed absorption signals at δ 68.8 ppm, corresponding to one carbon atom of the thiazole, 87.3 ppm, corresponding to one carbon atom of pyrimidine, 102.6 ppm, corresponding to one carbon atom of the phenyl, and 164.8, 167.9, and 172.9 ppm, corresponding to the three carbon atoms of the carbonyl groups. Furthermore, the refluxing of 12a or 12b with a suitable aromatic aldehyde, namely, benzaldehyde, 4-chloro- benzaldehyde, or 4-methoxybenzaldehyde respectively, in dioxane solution having a catalyst amount of piperidine for an extended time gave the products 2,10-di(substituted-benzylidene)- 8-((4-methoxy or 4,8-dimethoxy)-3-methylbenzo[1,2- b:5,4-b`]difuran-2-yl)-5H,12aH-thiazolo [2’, 3’: 2,3]pyrimido[6,1-d][1,3,5]oxadiazepine-3,5,11(2H,10H)- trione (13a–f) in high yields. In another way, we obtained the same products (13a–f) via reacting 12a or 12b with a proper aromatic aldehyde and anhydrous sodium acetate in glacial acetic acid with acetic anhydride for 8–10 h. The ^1^H NMR spectrum of **13a** displayed two singlets at 8.04 and 8.10 ppm conforming to the two methine protons (2CH). The mass spectra of **13a**, **13b**, **13c, 13d, 13e,** and **13f** exposed molecular ion peaks at *m/z* 629 (M^+^, 90%), 698 (M^+^, 92%), 689 (M^+^, 88%), 659 (M^+^, 88%), 728 (M^+^, 95%), and 719 (M^+^, 94%), respectively, All spectral analyses of new compounds are described in the Experimental section (Scheme 4).

### 2.2. Biological Screening

#### 2.2.1. The Inhibition Test of COX Enzymes in vitro

In Table 1, the IC_50_ results of COX inhibition and the selectivity index (SI) are shown. The evaluation results evidenced that all compounds displayed efficient inhibitory activities on COX-2 enzymes, with IC_50_ values that were lower than those of COX-1, in the range of 0.04–0.46 μM, compared to COX-2. Moreover, the inhibitory activity against COX-1 ranged from 4.15 to 18.80 μM. The compounds **10a**–**d**, **13a**–**f**, **12a**–**b,** and **11a**–**b** had the highest inhibitory activities with IC_50_ values of 4.15–4.60, 5.10–5.79, 6.10–6.40, and 6.60–6.85 μM to COX-1, respectively, and 0.042–0.048, 0.053–0.064, 0.070–0.075, and 0.080–0.085 μM to COX-2, respectively, indicating the highest selectivity index of 99–81 respectively. In addition, **10a**–**d**, **13a**–**f**, **12a**–**b**, **11a**–**b,** and **7a**–**b** compounds exhibited the highest COX-2 selectivity index.

#### 2.2.2. Analgesic Activity

Table 2 shows the results of the analgesic activity of the new compounds in vivo via applying the writhing test [4,30,31,32,33,34,35], with results evaluated by comparing them to the same dose (10 mg/kg) of the standard drug sodium diclofenac. The writhing response was calculated, and protection (%) was calculated. Compounds **10a**–**d**, **13a**–**f,** and **12a**–**b** evidenced the highest analgesic activities among the synthesized compounds, 48–51%, 42–47%, and 40–41% respectively, which were nearly the same activities of the drug sodium diclofenac (51%). In addition, the analgesic activities of the aforementioned compounds agreed with their anti-inflammatory activity. On the other hand, compounds **4a**–**b**, **5a**–**b,** and **6a**–**b** showed the lowest activities, which may be due to several pharmacokinetic parameters that affected the absorption and degradation of these compounds.

#### 2.2.3. Anti-Inflammatory Activity

From the past results, the determined compounds **10a**–**d**, **13a**–**f**, **12a**–**b**, **11a**–**b,** and **7a**–**b** possessed the highest anti-inflammatory activities. A carrageenan-induced paw edema in rats was used to evaluate the anti-inflammatory activity of these compounds [4,30,31,32,33,34,36]. Most of the compounds evidenced significant (*p* < 0.05) anti-inflammatory activity, via reducing paw height; hence, rat paw edema was reduced after three hours in comparison with the control group, as shown in Table 3. The compounds **10a**–**d**, **13a**–**f**, **12a**–**b**, and **11a**–**b,** among all the synthesized compounds, displayed the highest anti-inflammatory activity, which appeared from the first hour of inflammation with edema inhibition percentages of 86–65%, 64–59%, 58–57%, and 56–55%, respectively. This was superior to the drug sodium diclofenac, with a rapid onset of action after one hour, and the time continued until the third hour after compound administration.

#### 2.2.4. Serum Level of Interleukin-1 Beta (IL-1 β)

At the end of the paw edema test, serum was synthesized for ELISA to determine the interleukin-1 beta (IL-1 β) concentration in the most important groups. The determined compounds were **10a**–**d**, **13a**–**f**, **12a**–**b,** and **11a**–**b** (shown in Table 4). Compounds **10a–d** showed the most significant reduction in IL-1 β concentration, which established the in vitro inhibition of COX activity and so inhibited the inflammatory intermediator in this pathway.

#### 2.2.5. Structural Activity Relationship (SAR)

The analgesic and anti-inflammatory activity results indicated that the nucleus of piperazine, morpholine, pyrimidine, thiazole, and oxadiazepine moieties are important for the activity [4,32,33,34]. Thus, the compounds *N*-(2,6-di(piperazin-1-yl or morpholino) pyrimidin-4-yl)-benzodifuranyl-1- (piperazine or morpholine) methanimine **(10a**–**d)**, substituted benzylidene **(13a**–**f)**, 1,3,5- oxadiazepine **(12a,b)**, 1,3,5-triazine **(11a,b)**, and thiazolopyrimidine **(7a,b)** which linked with benzodifuranyl derivatives showed the highest activity through the inhibition of the enzyme cyclooxygenase (COX), which secretes prostaglandins. As well, the intermediate compounds such as *N*-(6-chloro-2-(thioxo or methylthio) pyrimidine)-benzodifuran-2-carbimidoyl chloride (**8a,b** and **9a,b**) are less active, indicating that certain structural features are necessary for the analgesic and anti-inflammatory activities. One the other hand, the compounds *N*-(6-oxo-2-thioxo-pyrimidine) benzodifuran-2-carboxamide (**5a, b** and **4a, b**) are showing weak activity because they are without a nucleus of the piperazine, morpholine, thiazole, and oxadiazepine and without some of the functional groups.

## 3. Experimental Section 

### 3.1. General Information

All melting points were taken on an Electrothermal IA 9100 series digital melting point apparatus (Shimadzu, Tokyo, Japan). Elemental analyses were performed on a Vario EL (Elementar, Langenselbold, Germany). Microanalytical data were processed in the microanalytical center, Faculty of Science, Cairo University and National Research Centre. The IR spectra (KBr disc) were recorded using a Perkin-Elmer 1650 spectrometer (Waltham, MA, USA). NMR spectra were determined using JEOL 270 MHz and JEOL JMS-AX 500 MHz (JEOL, Tokyo, Japan) spectrometers with Me4Si as an internal standard. Mass spectra were recorded on an EI Ms-QP 1000 EX instrument (Shimadzu, Japan) at 70 eV. Biological evaluations were done by the anti-inflammatory and analgesic activities unit, Mansoura University, Faculty of Pharmacy (Department of Pharmacognosy), 35516, Egypt. All starting materials and solvents were purchased from Sigma-Aldrich (Saint Louis, MO, USA).

### 3.2. General Procedure for Synthesis of 2-((5-acetyl-(4-methoxy or 4, 7-dimethoxy)-benzofuran -6-yl) oxy)-N-(6-oxo-2-thioxo-1,2,3,6-tetrahydropyrimidin-4-yl) acetamide *(**4a**, **b**)*

A solution of **2a** (2.92 g, 0.01 mol) or **2b** (3.22 g, 0.01 mol) with 6-amino-2-thioxo-2, 3-dihydropyrimidin-4 (1*H*)-one (1.43 g, 0.01 mol) and anhydrous potassium carbonate (0.015 mol) in dimethylformamide (50 mL) was refluxed for 6–8 h. The solid formed was filtered off, dried, and crystallized from the proper solvent to give **4a** and **4b**, respectively.

### 3.3. Synthesis of 2-((5-acetyl-4-methoxybenzofuran-6-yl) oxy)-N-(6-oxo-2-thioxo-1,2,3,6-tetrahydro pyrimidin-4-yl) acetamide *(**4a**)*

The compound was obtained from the reaction of **2a** with 6-aminothiouracil as yellowish crystals, which were crystallized from dioxane in 95% yield, m.p. 170–172 °C. IR (ν, cm^–1^) KBr: 3380 (brs, 3NH), 3050 (CH-aryl), 2970 (CH-aliph), 1730 (CO, acetyl), 1690 and 1680 (2CO, amide), and 1352 (C=S). ^1^H NMR (DMSO-*d*_6_, ppm) δ 2.30 (s, 3 H, CH_3_), 3.90 (s, 3 H, OCH_3_), 4.70 (s, 2 H, CH_2_), 6.70 (d, 1 H, J = 2.30 Hz, furan), 6.85 (s, 1 H, pyrimidine), 7.72 (d, 1 H, J = 2.32 Hz, furan), 7.80 (s, 1 H, phenyl), 8.50, 9.70, and 11.90 (br, 3 H, 3NH, D_2_O exchangeable). ^13^C NMR (DMSO-*d*_6_) δ 25.5 (1C, CH_3_), 60.1 (1C, OCH_3_), 70.3 (1C, CH_2_), 80.5 (1C, CH, pyrimidine), 94.4 (1C, CH, phenyl), 103.7, 107.2, 112.9, 142.5, 152.5, 154.2, 157.1, and 159.3 (8C, Ar-C), 162.5, 165.6, and 170.8 (3C, 3C=O), 178.9 (1C, C=S). MS (70 eV, %) *m*/*z* 389 (M^+^, 100%); Anal. Calc. (Found) for C_17_H_15_N_3_O_6_S (389.38): C, 52.44 (52.35); H, 3.88 (3.80); and N, 10.79 (10.85).

### 3.4. Synthesis of 2-((5-acetyl-4, 7-dimethoxybenzofuran-6-yl) oxy)-N-(6-oxo-2-thioxo-1,2,3,6-tetrahydro pyrimidin-4-yl) acetamide *(**4b**)*

The compound was obtained from the reaction of **2b** with 6-aminothiouracil as yellow crystals, which were crystallized from benzene in 92% yield, m.p. 186–188 °C. IR (*ν*, cm^–1^) KBr: 3382 (brs, 3NH), 3054 (CH-aryl), 2971 (CH-aliph), 1735 (CO, acetyl), 1692 and 1683 (2CO, amide), and 1355 (C=S). ^1^H NMR (DMSO-*d*_6_, ppm) *δ* 2.35 (s, 3 H, CH_3_), 3.94 (s, 6 H, 2OCH_3_), 4.75 (s, 2 H, CH_2_), 6.73 (d, 1 H, *J* = 2.33 Hz, furan), 6.88 (s, 1 H, pyrimidine), 7.75 (d, 1 H, *J* = 2.34 Hz, furan), 8.60, 9.75, and 11.95 (br, 3 H, 3NH, D_2_O exchangeable). ^13^C NMR (DMSO-*d*_6_) *δ* 25.8 (1C, CH_3_), 60.5 (2C, 2OCH_3_), 70.6 (1C, CH_2_), 80.7 (1C, CH, pyrimidine), 103.9, 107.8, 112.5, 135.3, 142.1, 145.6, 150.2, 152.4, and 157.5 (9C, Ar-C), 162.8, 165.9, and 171.2 (3C, 3C=O), 179.2 (1C, C=S). MS (70 eV, %) *m/z* 419 (M^+^, 100%); Anal. Calc. (Found) for C_18_H_17_N_3_O_7_S (419.41): C, 51.55 (51.50); H, 4.09 (4.15); and N, 10.02 (10.10).

### 3.5. General Procedure for Synthesis of (4-methoxy or 4, 8-dimethoxy)-3-methyl-N-(6-oxo-2-thioxo-1,2,3,6-tetrahydropyrimidin-4-yl) benzo [1,2-b: 5,4-b`] difuran-2-carboxamide *(**5a**, **b**)*

Method A: A mix of **2a** (2.92 g, 0.01 mol) or **2b** (3.22 g, 0.01 mol) with 6-amino-2-thiouracil (1.43 g, 0.01 mol) and anhydrous potassium carbonate (0.015 mol) in DMF (45 mL) was refluxed for 15–17 h. The final product precipitated was filtered off and washed with (100 mL) water, dried, and crystallized from the proper solvent to give **5a** and **5b**. Method B: A solution of compound 4a (3.89 g, 0.01 mol) or 4b (4.19g, 0.01 mol) in sodium ethoxide solution (prepared by dissolving 0.23 g of sodium metal in 40 mL ethanol) was heated under reflux with stirring for 5–7 h under control (TLC). The reaction mixture was allowed to cool and poured into cold water (100 mL) and neutralized via hydrochloric acid. The solid product was precipitated, which was filtered off and crystallized from the proper solvent to give **5a** and **5b**, respectively.

### 3.6. Synthesis of 4-methoxy-3-methyl-N-(6-oxo-2-thioxo-1,2,3,6-tetrahydropyrimidin-4-yl) benzo [1,2-b: 5, 4- b`] difuran-2-carboxamide *(**5a**)*

The compound was obtained from the reaction of **2a** with 6-aminothiouracil or compound (**4a)** as white crystals, which were crystallized from benzene in 88% yield, m.p. 245–247 °C. IR (*ν*, cm^–1^) KBr: 3390 (brs, 3NH), 3060 (CH-aryl), 2965 (CH-aliph), 1687 and 1683 (2CO, amide), and 1350 (C=S). ^1^H NMR (DMSO-*d*_6_, ppm) *δ* 2.38 (s, 3 H, CH_3_), 3.85 (s, 3 H, OCH_3_), 6.66 (d, 1 H, *J* = 2.37 Hz, furan), 6.80 (s, 1 H, pyrimidine), 7.20 (s, 1 H, phenyl), 7.65 (d, 1 H, *J* = 2.36 Hz, furan), 8.60, 9.80, and 12.10 (br, 3 H, 3NH, D_2_O exchangeable). ^13^C NMR (DMSO-*d*_6_) *δ* 18.6 (1C, CH_3_), 62.3 (1C, OCH_3_), 83.1 (1C, CH, pyrimidine), 101.5 (1C, CH, phenyl), 104.7, 106.8, 118.5, 123.4, 127.6, 144.3, 146.9, 156.4, 157.8, and 159.9 (10C, Ar-C), 163.2 and 168.5 (2C, 2C=O), 179.4 (1C, C=S). MS (70 eV, %) *m/z* 371 (M^+^, 100%); Anal. Calc. (Found) for C_17_H_13_N_3_O_5_S (371.37): C, 54.98 (54.90); H, 3.53 (3.61); and N, 11.32 (11.40).

### 3.7. Synthesis of 4, 8-dimethoxy-3-methyl-N-(6-oxo-2-thioxo-1,2,3,6-tetrahydropyrimidin-4-yl) benzo [1,2-b: 5, 4- b`] difuran-2-carboxamide *(**5b**)*

The compound was obtained from the reaction of **2b** with 6-aminothiouracil or compound (**4b)** as yellowish crystals, which were crystallized from dioxane in 85% yield, m. p. 266–268 °C. IR (*ν*, cm^–1^) KBr: 3395 (brs, 3NH), 3055 (CH-aryl), 2962 (CH-aliph), 1685 and 1680 (2CO, amide), and 1348 (C=S). ^1^H NMR (DMSO-*d*_6_, ppm) *δ* 2.36 (s, 3 H, CH_3_), 3.80 (s, 6 H, 2OCH_3_), 6.69 (d, 1 H, *J* = 2.38 Hz, furan), 6.85 (s, 1H, pyrimidine), 7.68 (d, 1 H, *J* = 2.35 Hz, furan), 8.64, 9.88, and 12.15 (br, 3 H, 3NH, D_2_O exchangeable). ^13^C NMR (DMSO-*d*_6_) *δ* 18.9 (1C, CH_3_), 64.8 (2C, 2OCH_3_), 84.3 (1C, CH, pyrimidine), 105.4, 106.6, 116.5, 118.8, 122.9, 138.2, 145.7, 146.8, 147.9, 148.7, and 159.4 (11C, Ar-C), 162.8 and 168.7 (2C, 2C=O), 179.6 (1C, C=S). MS (70 eV, %) *m/z* 401 (M^+^, 100%); Anal. Calc. (Found) for C_18_H_15_N_3_O_6_S (401.39): C, 53.86 (53.75); H, 3.77 (3.70); and N, 10.47 (10.55).

### 3.8. General Procedure for Synthesis of 2-((4-((4-methoxy or 4, 8-dimethoxy)-3-methylbenzo [1,2-b: 5, 4- b`] difuran-2-carboxamido)-6-oxo-1, 6-dihydropyrimidin-2-yl) thio) acetic acid *(**6a**, **b**)*

A mix of **5a** (3.71 g, 0.01 mol) or **5b** (4.01 g, 0.01 mol), chloroacetic acid (0.94 g, 0.01 mol), and (0.02 mol) anhydrous sodium acetate was stirred under reflux in 45 mL of glacial acetic acid and 25 mL of acetic anhydride for 4–6 h. The reaction solution was cooled and poured into cold water (100 mL). The deposited precipitate was filtered off and crystallized from suitable solvent to yield **6a** and **6b**, respectively.

### 3.9. Synthesis of 2-((4-(4-methoxy-3-methylbenzo [1,2-b: 5,4-b`] difuran-2-carboxamido)-6-oxo-1, 6-dihydro pyrimidin-2-yl) thio) acetic acid *(**6a**)*

The compound was obtained from the reaction of **5a** with chloroacetic acid as brownish crystals, which were crystallized from methanol in 82% yield, m.p. 278–280 °C. IR (*ν*, cm^–1^) KBr: 3380 (brs, 2NH), 3350 (br, OH), 3052 (CH-aryl), 2955 (CH-aliph), 1750 (CO, acid), 1685 and 1680 (2CO, amide), and 1630 (C=N). ^1^H NMR (DMSO-*d*_6_, ppm) *δ* 2.32 (s, 3 H, CH_3_), 3.95 (s, 3 H, OCH_3_), 4.10 (s, 2 H, CH_2_), 6.70 (d, 1 H, *J* = 2.31 Hz, furan), 6.88 (s, 1 H, pyrimidine), 7.25 (s, 1H, phenyl), 7.64 (d, 1 H, *J* = 2.33 Hz, furan), 10.10 and 11.95 (br, 2 H, 2NH, D_2_O exchangeable), and 12.50 (br, 1 H, OH, D_2_O exchangeable). ^13^C NMR (DMSO-*d*_6_) *δ* 18.9 (1C, CH_3_), 31.6 (1C, CH_2_), 60.8 (1C, OCH_3_), 84.2 (1C, CH, pyrimidine), 102.3 (1C, CH, phenyl), 104.5, 106.3, 118.2, 123.1, 127.3, 144.8, 146.5, 152.7, 155.9, 158.1, and 160.2 (11C, Ar-C), 162.4, 166.1, and 172.3 (3C, 3C=O). MS (70 eV, %) *m/z* 429 (M^+^, 100%); Anal. Calc. (Found) for C_19_H_15_N_3_O_7_S (429.40): C, 53.15 (53.25); H, 3.52 (3.45); and N, 9.79 (9.84).

### 3.10. Synthesis of 2-((4-(4, 8-dimethoxy-3-methylbenzo [1,2-b: 5,4-b`] difuran-2-carboxamido)-6-oxo-1, 6- dihydropyrimidin-2-yl) thio) acetic acid *(**6b**)*

The compound was obtained from the reaction of **5b** with chloroacetic acid as yellowish crystals, which were crystallized from toluene in 84% yield, m.p. 293–295 °C. IR (*ν*, cm^–1^) KBr: 3382 (brs, 2NH), 3354 (br, OH), 3051 (CH-aryl), 2953 (CH-aliph), 1753 (CO, acid), 1687 and 1681 (2CO, amide), and 1633 (C=N). ^1^H NMR (DMSO-*d*_6_, ppm) *δ* 2.34 (s, 3 H, CH_3_), 3.97 (s, 6 H, 2OCH_3_), 4.15 (s, 2 H, CH_2_), 6.71 (d, 1 H, *J* = 2.33 Hz, furan), 6.90 (s, 1 H, pyrimidine), 7.68 (d, 1 H, *J* = 2.35 Hz, furan), 10.20 and 11.90 (br, 2 H, 2NH, D_2_O exchangeable), and 12.55 (br, 1 H, OH, D_2_O exchangeable). ^13^C NMR (DMSO-*d*_6_) *δ* 19.1 (1C, CH_3_), 31.9 (1C, CH_2_), 61.2 (2C, 2OCH_3_), 84.5 (1C, CH, pyrimidine), 105.4, 106.6, 117.1, 118.8, 122.9, 138.2, 145.5, 146.3, 147.6, 148.1, 154.5, and 161.3 (12C, Ar-C), 162.9, 166.9, and 172.8 (3C, 3C=O). MS (70 eV, %) *m/z* 459 (M^+^, 100%); Anal. Calc. (Found) for C_20_H_17_N_3_O_8_S (459.43): C, 52.29 (52.35); H, 3.73 (3.80); and N, 9.15 (9.25).

### 3.11. General Procedure for Synthesis of N-(3,5-dioxo-2,3-dihydro-5H-thiazolo[3,2-a]pyrimidin-7-yl)-(4- methoxy or 4,8-dimethoxy)-3-methylbenzo[1,2-b: 5,4-b`]difuran-2-carboxamide *(**7a**, **b**)*

**Method A:** A mix of compounds **5a** (3.71 g, 0.01 mol) or **5b** (4.01 g, 0.01 mol), chloroacetic acid (0.94 g, 0.01 mol), and 0.02 mol of anhydrous sodium acetate was stirred under reflux in 45 mL of glacial acetic acid and 25 mL of acetic anhydride in a water bath (80–90 °C) for 14–16 h under TLC. The reaction mix was allowed to cool to room temperature and poured into H_2_O (100 mL). The final solid precipitate was filtered off and crystallized from suitable solvent to form **7a** and **7b** in good yields, respectively. **Method B:** A solution of **6a** (4.29g, 0.01 mol) or **6b** (4.59g, 0.01 mol) in dimethylformamide (40 mL) was refluxed for 8–10 h under control (TLC). The final product was filtered off, dried, and crystallized from a suitable solvent to give **7a** and **7b**, respectively.

### 3.12. Synthesis of N-(3,5-dioxo-2, 3-dihydro-5H-thiazolo [3, 2-a] pyrimidin-7-yl)-4-methoxy-3-methylbenzo [1,2-b: 5,4-b`] difuran-2-carboxamide *(**7a**)*

The compound was obtained from the reaction of **5a** with chloroacetic acid or refluxed **(6a)** in DMF as yellowish crystals, which were crystallized from ethanol in 84% yield, m.p. 308–310 °C. IR (*ν*, cm^–1^) KBr: 3340 (br, NH), 3050 (CH-aryl), 2950 (CH-aliph), 1685, 1680, and 1675 (3CO, amide), 1635 (C=N). ^1^H NMR (DMSO-*d*_6_, ppm) *δ* 2.38 (s, 3 H, CH_3_), 4.01 (s, 3 H, OCH_3_), 4.20 (s, 2 H, CH_2_), 6.72 (d, 1 H, *J* = 2.34 Hz, furan), 6.95 (s, 1 H, pyrimidine), 7.05 (s, 1H, phenyl), 7.68 (d, 1 H, *J* = 2.39 Hz, furan), and 10.20 (br, 1H, NH, D_2_O exchangeable). ^13^C NMR (DMSO-*d*_6_) *δ* 19.2 (1C, CH_3_), 30.2 (1C, CH_2_), 62.3 (1C, OCH_3_), 86.5 (1C, CH, pyrimidine), 102.7 (1C, CH, phenyl), 104.8, 106.2, 118.6, 123.5, 127.5, 145.2, 146.7, 154.5, 155.6, 158.9, and 159.4 (11C, Ar-C), 163.1, 165.4, and 173.5 (3C, 3C=O). MS (70 eV, %) *m/z* 411 (M^+^, 100%); Anal. Calc. (Found) for C_19_H_13_N_3_O_6_S (411.39): C, 55.47 (55.40); H, 3.19 (3.12); and N, 10.21 (10.30).

### 3.13. Synthesis of N-(3,5-dioxo-2,3-dihydro-5H-thiazolo[3,2-a]pyrimidin-7-yl)-4,8-dimethoxy-3- methylbenzo [1,2-b:5,4-b`]difuran-2-carboxamide *(**7b**)*

The compound was obtained from the reaction of **5b** with chloroacetic acid or refluxed (**6b**) in DMF as yellow crystals, which were crystallized from methanol in 80% yield, m.p. 327–329 °C. IR (*ν*, cm^–1^) KBr: 3345 (br, NH), 3051 (CH-aryl), 2954 (CH-aliph), 1687, 1683, and 1678 (3CO, amide), 1634 (C=N). ^1^H NMR (DMSO-*d*_6_, ppm) *δ* 2.39 (s, 3 H, CH_3_), 4.10 (s, 6 H, 2OCH_3_), 4.25 (s, 2 H, CH_2_), 6.73 (d, 1 H, *J* = 2.30 Hz, furan), 6.98 (s, 1 H, pyrimidine), 7.70 (d, 1 H, *J* = 2.31 Hz, furan), and 10.28 (br, 1H, NH, D_2_O exchangeable). ^13^C NMR (DMSO-*d*_6_) *δ* 19.4 (1C, CH_3_), 30.5 (1C, CH_2_), 64.1 (2C, 2OCH_3_), 86.8 (1C, CH, pyrimidine), 105.5, 106.7, 115.9, 118.8, 123.1, 137.9, 145.5, 146.4, 147.6, 147.9, 154.2, and 159.7 (12C, Ar-C), 163.5, 165.9, and 173.8 (3C, 3C=O). MS (70 eV, %) *m/z* 441 (M^+^, 100%); Anal. Calc. (Found) for C_20_H_15_N_3_O_7_S (441.41): C, 54.42 (54.50); H, 3.43 (3.51); and N, 9.52 (9.60).

### 3.14. General Procedure for Synthesis of N-(6-chloro-2-thioxo-2, 3-dihydropyrimidin-4-yl)-(4-methoxy or 4, 8-dimethoxy)-3-methyl benzo [1,2-b: 5,4-b`] difuran-2-carbimidoyl chloride *(**8a**, **b**)*

A solution of **5a** (3.71 g, 0.01 mol) or **5b** (4.01 g, 0.01 mol) in dry dioxane (30 mL) was treated with 20 mL of phosphorus oxy-chloride, and the mixture was stirred under reflux for 4–6 h. The reaction mixture was allowed to cool to room temperature. Then, it was poured into cold water (100 mL), whereby a solid was separated, filtered off, and crystallized from a suitable solvent to give **8a** and **8b**, respectively.

### 3.15. Synthesis of N-(6-chloro-2-thioxo-2, 3-dihydropyrimidin-4-yl)-4-methoxy-3-methylbenzo [1,2-b: 5,4-b`] difuran-2-carbimidoyl chloride *(**8a**)*

The compound was obtained from the reaction of **5a** with phosphorus oxy-chloride as yellowish crystals, which were crystallized from dioxane in 85% yield, m.p. 337–339 °C. IR (*ν*, cm^–1^) KBr: 3360 (br., NH), 3050 (CH-aryl), 2945 (CH-aliph), 1636 (C=N), and 1358 (C=S). ^1^H NMR (DMSO-*d*_6_, ppm) *δ* 2.29 (s, 3 H, CH_3_), 3.83 (s, 3 H, OCH_3_), 6.62 (d, 1 H, *J* = 2.30 Hz, furan), 6.75 (s, 1 H, pyrimidine), 7.24 (s, 1 H, phenyl), 7.60 (d, 1 H, *J* = 2.32 Hz, furan), and 12.70 (br., 1 H, NH, D_2_O exchangeable). ^13^C NMR (DMSO-*d*_6_) *δ* 18.9 (1C, CH_3_), 63.1 (1C, OCH_3_), 87.2 (1C, CH, pyrimidine), 102.1 (1C, CH, phenyl), 104.4, 106.3, 117.6, 118.2, 125.7, 130.5, 146.2, 150.3, 150.8, 156.1, 157.9, and 161.2 (12C, Ar-C), 182.1 (1C,C=S). MS (70 eV, %) *m/z* 408 (M^+^, 90%); Anal. Calc. (Found) for C_17_H_11_Cl_2_N_3_O_3_S (408.25): C, 50.01 (50.15); H, 2.72 (2.65); and N, 10.29 (10.35).

### 3.16. Synthesis of N-(6-chloro-2-thioxo-2, 3-dihydropyrimidin-4-yl)-4, 8-dimethoxy-3-methylbenzo [1,2-b: 5, 4- b`] difuran-2-carbimidoyl chloride *(**8b**)*

The compound was obtained from the reaction of **5b** with phosphorus oxy-chloride as yellow crystals, which were crystallized from methanol in 82% yield, m.p. 347–349 °C. IR (*ν*, cm^–1^) KBr: 3362 (br., NH), 3054 (CH-aryl), 2948 (CH-aliph), 1638 (C=N), and 1357 (C=S). ^1^H NMR (DMSO-*d*_6_, ppm) *δ* 2.28 (s, 3 H, CH_3_), 3.88 (s, 6 H, 2OCH_3_), 6.66 (d, 1 H, *J* = 2.31 Hz, furan), 6.72 (s, 1 H, pyrimidine), 7.64 (d, 1 H, *J* = 2.33 Hz, furan), and 12.75 (br., 1H, NH, D_2_O exchangeable). ^13^C NMR (DMSO-*d*_6_) *δ* 19.1 (1C, CH_3_), 65.4 (2C, 2OCH_3_), 87.5 (1C, CH, pyrimidine), 105.3, 106.6, 116.8, 118.5, 118.7, 130.8, 1138.1, 146.1, 147.3, 147.8, 150.4, 150.9, and 161.5 (13C, Ar-C), and 182.3 (1C, C=S). MS (70 eV, %) *m/z* 438 (M^+^, 92%); Anal. Calc. (Found) for C_18_H_13_Cl_2_N_3_O_4_S (438.28): C, 49.33 (49.41); H, 2.99 (2.91); and N, 9.59 (9.65).

### 3.17. General Procedure for Synthesis of N-(6-chloro-2-(methylthio) pyrimidin-4-yl)-(4-methoxy or 4, 8-dimethoxy)-3-methylbenzo [1,2-b: 5, 4- b`] difuran-2-carbimidoyl chloride *(**9a**, **b**)*

To a warmed ethanolic potassium hydroxide solution (prepared by dissolving 10 mmol of KOH in 50 mL ethanol), **8a** (4.08 g, 0.01 mol) or **8b** (4.38 g, 0.01 mol) was added, and the heating was continued for 35 min. Then, the mixture was allowed to cool to room temperature, and methyl iodide (0.62 mL, 0.01 mol) was added. The mixture was stirred under reflux for 6–8 h; then, it was cooled to room temperature and poured into cold water (100 mL). The final solid precipitated was filtered off, washed with water, and the product was dried and crystallized from a suitable solvent to yield **9a** and **9b**, respectively.

### 3.18. Synthesis of N-(6-chloro-2-(methylthio) pyrimidin-4-yl)-4-methoxy-3-methylbenzo [1,2-b: 5,4-b`] difuran-2-carbimidoyl chloride *(**9a**)*

The compound was obtained from the reaction of **8a** with methyl iodide as brownish crystals, which were crystallized from DMF in 80% yield, m.p. >350 °C. IR (*ν*, cm^–1^) KBr: 3044 (CH-aryl), 2940 (CH-aliph), and 1634 (C=N). ^1^H NMR (DMSO-*d*_6_, ppm) *δ* 2.25 (s, 3 H, SCH_3_), 2.30 (s, 3 H, CH_3_), 3.87 (s, 3 H, OCH_3_), 6.68 (d, 1 H, *J* = 2.33 Hz, furan), 6.77 (s, 1 H, pyrimidine), 7.20 (s, 1 H, phenyl), and 7.64 (d, 1 H, *J* = 2.37 Hz, furan). ^13^C NMR (DMSO-*d*_6_) *δ* 19.3 (1C, CH_3_), 22.8 (1C, SCH_3_), 65.6 (1C, OCH_3_), 101.4 (1C, CH, phenyl), 103.5 (1C, CH, pyrimidine), 105.6, 106.8, 117.4, 118.3, 126.2, 130.9, 146.5, 150.7, 155.6, 157.9, 161.9, 167.2, and 171.5 (13C, Ar-C). MS (70 eV, %) *m/z* 422 (M^+^, 94%); Anal. Calc. (Found) for C_18_H_13_Cl_2_N_3_O_3_S (422.28): C, 51.20 (51.30); H, 3.10 (3.18); and N, 9.95 (9.88).

### 3.19. Synthesis of N-(6-chloro-2-(methylthio) pyrimidin-4-yl)-4, 8-dimethoxy-3-methylbenzo [1,2-b: 5,4-b`] difuran-2-carbimidoyl chloride *(**9b**)*

The compound was obtained from the reaction of **8b** with methyl iodide as brown crystals, which were crystallized from dioxane in 82% yield, m.p. >350 °C. IR (*ν*, cm^–1^) KBr: 3047 (CH-aryl), 2943 (CH-aliph), and 1632 (C=N). ^1^H NMR (DMSO-*d*_6_, ppm) *δ* 2.23 (s, 3 H, SCH_3_), 2.33 (s, 3 H, CH_3_), 3.91 (s, 6 H, OCH_3_), 6.62 (d, 1 H, *J* = 2.35 Hz, furan), 6.82 (s, 1 H, pyrimidine), and 7.70 (d, 1 H, *J* = 2.39 Hz, furan). ^13^C NMR (DMSO-*d*_6_) *δ* 19.1 (1C, CH_3_), 22.5 (1C, SCH_3_), 65.2 (2C, 2OCH_3_), 102.8 (1C, CH, pyrimidine), 105.4, 106.2, 115.6, 117.8, 118.5, 131.3, 137.9, 146.7, 147.5, 148.2, 150.7, 161.3, 168.5, and 172.1 (14C, Ar-C). MS (70 eV, %) *m/z* 452 (M^+^, 90%); Anal. Calc. (Found) for C_19_H_15_Cl_2_N_3_O_4_S (452.31): C, 50.45 (50.52); H, 3.34 (3.41); and N, 9.29 (9.35).

### 3.20. General Procedure for Synthesis of N-(2, 6-di (piperazin-1-yl or morpholino) pyrimidin-4-yl)-1-((4- methoxy or 4, 8-dimethoxy)-3-methylbenzo [1,2-b: 5, 4- b`] difuran-2-yl)-1-(piperazin-1-yl or morpholino) methanimine *(**10a**–**d**)*

In a warm solution of **9a** (4.22 g, 0.01 mol) or **9b** (4.52 g, 0.01 mol) in glacial acetic acid (40 mL) or absolute methanol (40 mL), freshly distilled secondary (2°) amines, piperazine (0. 86 g, 0.01 mol), or morpholine (0.87 mL, 0.01 mol) were added. The reaction mixture was stirred under reflux for 5–7 h with TLC; then, it was allowed to cool to 0 °C for 4 h. The solid obtained was filtered, washed with water (100 mL), dried, and recrystallized from an appropriate solvent to produce **10a–d**.

### 3.21. Synthesis of N-(2, 6-di (piperazin-1-yl) pyrimidin-4-yl)-1-(4-methoxy-3-methylbenzo [1,2-b: 5,4-b`] difuran-2-yl)-1-(piperazin-1-yl) methanimine *(**10a**)*

The compound was obtained from the reaction of **9a** with piperazine as yellowish crystals, which were crystallized from dioxane in 78% yield, m.p. >350 °C. IR (*ν*, cm^–1^) KBr: 3385 (br, NHs), 3050 (CH-aryl), 2937 (CH-aliph), and 1630 (C=N). ^1^H NMR (DMSO-*d*_6_, ppm) *δ* 2.28 (s, 3 H, CH_3_), 2.70–2.82 (m, 12H, piperazine), 3.13–3.25 (m, 12H, piperazine), 3.85 (s, 3 H, OCH_3_), 6.10 (s, 1 H, pyrimidine), 6.72 (d, 1 H, *J* = 2.35 Hz, furan), 7.05 (s, 1 H, phenyl), 7.66 (d, 1 H, *J* = 2.34 Hz, furan), 10.10, 10.30, and 10.50 (br, 3NH, D_2_O exchangeable). ^13^C NMR (DMSO-*d*_6_) *δ* 20.5 (1C,CH_3_), 46.5, 48.8, 50.4, 52.6, 53.9, and 55.5 (12C, three piperazine rings), 63.8 (1C, OCH_3_), 90.5 (1C, CH, pyrimidine), 101.9 (1C, CH, phenyl), 104.5, 106.2, 117.7, 118.5, 126.8, 131.2, 146.3, 154.8, 157.2, 161.9, 162.3, 168.8, and 173.7 (13C, Ar-C). MS (70 eV, %) *m/z* 559 (M^+^, 92%); Anal. Calc. (Found) for C_29_H_37_N_9_O_3_ (559.67): C, 62.24 (62.33); H, 6.66 (6.73); and N, 22.52 (22.45).

### 3.22. Synthesis of N-(2, 6-dimorpholinopyrimidin-4-yl)-1-(4-methoxy-3-methylbenzo [1,2-b: 5,4-b`] difuran-2-yl)-1-morpholinomethanimine *(**10b**)*

The compound was obtained from the reaction of **9a** with morpholine as yellow crystals, which were crystallized from methanol in 75% yield, m.p. >350 °C. IR (*ν*, cm^–1^) KBr: 3052 (CH-aryl), 2940 (CH-aliph), and 1632 (C=N). ^1^H NMR (DMSO-*d*_6_, ppm) *δ* 2.25 (s, 3 H, CH_3_), 3.20–3.32 (m, 12H, morpholine), 3.65–3.76 (m, 12H, morpholine), 3.87 (s, 3 H, OCH_3_), 6.20 (s, 1 H, pyrimidine), 6.74 (d, 1 H, *J* = 2.32 Hz, furan), 7.12 (s, 1 H, phenyl), and 7.70 (d, 1 H, *J* = 2.36 Hz, furan). ^13^C NMR (DMSO-*d*_6_) *δ* 21.1 (1C, CH_3_), 48.8, 49.9, 50.3, 58.9, 59.7, and 60.8 (12C, three morpholine rings), 64.1 (1C, OCH_3_), 91.2 (1C, CH, pyrimidine), 102.3 (1C, CH, phenyl), 104.3, 106.5, 117.9, 118.8, 127.1, 131.7, 146.5, 155.4, 157.8, 162.5, 163.6, 169.7, and 174.1 (13C, Ar-C). MS (70 eV, %) *m/z* 562 (M^+^, 90%); Anal. Calc. (Found) for C_29_H_34_N_6_O_6_ (562.63): C, 61.91 (61.82); H, 6.09 (6.15); and N, 14.94 (14.85).

### 3.23. Synthesis of N-(2, 6-di (piperazin-1-yl) pyrimidin-4-yl)-1-(4, 8-dimethoxy-3-methylbenzo [1,2-b: 5,4-b`] difuran-2-yl)-1-(piperazin-1-yl) methanimine *(**10c**)*

The compound was obtained from the reaction of **9b** with piperazine, as brownish crystals, which were crystallized from DMF in 73% yield, m.p. >350 °C. IR (*ν*, cm^–1^) KBr: 3390 (br, NHs), 3054 (CH-aryl), 2942 (CH-aliph), and 1628 (C=N). ^1^H NMR (DMSO-*d*_6_, ppm) *δ* 2.26 (s, 3 H, CH_3_), 2.73–2.85 (m, 12H, piperazine), 3.17–3.29 (m, 12H, piperazine), 4.05 (s, 6H, 2OCH_3_), 6.28 (s, 1 H, pyrimidine), 6.74 (d, 1 H, *J* = 2.31 Hz, furan), 7.68 (d, 1 H, *J* = 2.38 Hz, furan), 10.20, 10.40, and 10.60 (br, 3NH, D_2_O exchangeable). ^13^C NMR (DMSO-*d*_6_) *δ* 20.8 (1C, CH_3_), 46.8, 48.6, 50.7, 52.3, 53.7, and 55.8 (12C, three piperazine ring)s, 65.5 (2C, 2OCH_3_), 90.7 (1C, CH, pyrimidine), 105.6, 106.4, 116.7, 118.3, 118.8, 130.7, 137.9, 146.5, 147.3, 147.8, 162.2, 162.5, 169.1, and 174.3 (14C, Ar-C). MS (70 eV, %) *m/z* 589 (M^+^, 94%); Anal. Calc. (Found) for C_30_H_39_N_9_O_4_ (589.70): C, 61.10 (61.25); H, 6.67 (6.75); and N, 21.38 (21.30).

### 3.24. Synthesis of 1-(4, 8-dimethoxy-3-methylbenzo [1,2-b: 5, 4- b`] difuran-2-yl)-N-(2, 6-dimorpholino- pyrimidin-4-yl)-1-morpholinomethanimine *(**10d**)*

The compound was obtained from the reaction of **9b** with morpholine, as brown crystals, which were crystallized from THF in 70% yield, m.p. >350 °C. IR (*ν*, cm^–1^) KBr: 3056 (CH-aryl), 2945 (CH-aliph), and 1629 (C=N). ^1^H NMR (DMSO-*d*_6_, ppm) *δ* 2.24 (s, 3 H, CH_3_), 3.15–3.27 (m, 12H, morpholine), 3.70–3.81 (m, 12H, morpholine), 3.90 (s, 6H, 2OCH_3_), 6.28 (s, 1 H, pyrimidine), 6.78 (d, 1 H, *J* = 2.36 Hz, furan), and 7.74 (d, 1 H, *J* = 2.38 Hz, furan). ^13^C NMR (DMSO-*d*_6_) *δ* 21.5 (1C, CH_3_), 47.6, 48.4, 49.5, 57.3, 58.2, and 60.1 (12C, three morpholine rings), 65.2 (2C, 2OCH_3_), 91.8 (1C, CH, pyrimidine), 105.6, 106.9, 116.2, 118.2, 118.6, 130.4, 138.1, 146.3, 147.5, 147.9, 162.2, 162.8, 170.1, and 174.7 (14C, Ar-C). MS (70 eV, %) *m/z* 592 (M^+^, 85%); Anal. Calc. (Found) for C_30_H_36_N_6_O_7_ (592.65): C, 60.80 (60.72); H, 6.12 (6.22); and N, 14.18 (14.25).

### 3.25. General Procedure for Synthesis of 8-(4-methoxy or 4,8-dimethoxy-3-methylbenzo[1,2-b:5,4- b`] difuran- 2-yl)-2-hydro-5H,11aH-thiazolo[2’,3’:2,3]pyrimido[1,6-a][1,3,5]triazine-3,5-dione (11a,b) and Synthesis of 8-(4-methoxy or 4,8-dimethoxy-3-methylbenzo[1,2-b:5,4- b`]difuran-2-yl)-5H,12aH-thiazolo [2’,3’:2,3] pyrimido[6,1-d][1,3,5]oxadiazepine-3,5,11(2H,10H)-trione *(**12a**, **b**)*

Method A: A mixture of compound **7a** (4.11 g, 0.01 mol) or **7b** (4.41 g, 0.01 mol) and formamide (10 mL) or chloro-acetyl-chloride (1.13 g, 0.01 mol) was stirred under reflux in dimethylformamide (40 mL) in the presence of anhydrous potassium carbonate (0.015 mol) for 18–20 h under control (TLC). The reaction mixture was allowed to cool to room temperature, poured into water (100 mL), and neutralized. The solid formed was filtered off, dried, and crystallized from appropriate solvent to produce **11a**, **b** and **12a**, **b** respectively. Method B: The stirring of mixture of compound **7a** (4.11 g, 0.01 mol) or **7b** (4.41 g, 0.01 mol) and formamide (10 mL) or chloro-acetyl-chloride (1.13 g, 0.01 mol) in glacial acetic acid (45 mL), activated zinc dust (1.3 g, 0.02 mol) was added portionwise at room temperature over a period of 1 h. Stirring was continued for an additional 3–5 h. The reaction mixture was heated on a water bath (85–95 °C) for 4-6 h under control (TLC). After allowing the reaction mixture to cool to room temperature, it was poured into cold water (100 mL). The precipitate was filtered, washed with water, dried, and crystallized to produce **11a**, **b** and **12a**, **b** respectively.

### 3.26. Synthesis of 8-(4-methoxy-3-methylbenzo[1,2-b:5,4- b`]difuran-2-yl)-2-hydro-5H,11aH-thiazolo[2’,3’: 2,3]pyrimido[1,6-a][1,3,5]triazine-3,5-dione *(**11a**)*

The compound was obtained from the reaction of **7a** with formamide in DMF as pale yellow crystals, which were crystallized from benzene in 91% yield, m.p. >350 °C. IR (*ν*, cm^–1^) KBr: 3065 (CH-aryl), 2940 (CH-aliph), 1684 and 1680 (2CO, amide), and 1632 (C=N). ^1^H NMR (DMSO-*d*_6_, ppm) *δ* 2.32 (s, 3 H, CH_3_), 3.94 (s, 3 H, OCH_3_), 4.10 (d, 1 H, *J* = 6.90 Hz, CH_2,_ thiazole), 4.17 (d, 1 H, *J* = 6.91 Hz, CH_2,_ thiazole), 5.85 (s, 1 H, thiazole), 6.70 (d, 1 H, *J* = 2.31 Hz, furan), 7.20 (s, 1H, phenyl), 7.35 (s, 1H, triazine), 7.73 (d, 1 H, *J* = 2.37 Hz, furan), and 8.10 (s, 1 H, pyrimidine). ^13^C NMR (DMSO-*d*_6_) *δ* 21.5 (1C, CH_3_), 33.4 (1C, CH_2_), 61.7 (1C, OCH_3_), 70.10 (1C, CH, thiazole), 88.2 (1C, CH, pyrimidine), 102.4 (1C, CH, phenyl), 104.5, 106.7, 118.1, 118.7, 126.9, 130.5, 142.3, 146.8, 155.8, 158.1, 162.5, and 163.1 (12C, Ar-C), 166.7 and 169.8 (2C, 2C=O). MS (70 eV, %) *m/z* 422 (M^+^, 95%); Anal. Calc. (Found) for C_20_H_14_N_4_O_5_S (422.41): C, 56.87 (56.94); H, 3.34 (3.42); and N, 13.26 (13.33).

### 3.27. Synthesis of 8-(4,8-dimethoxy-3-methylbenzo[1,2-b:5,4- b`]difuran-2-yl)-2-hydro-5H,11aH-thiazolo [2’, 3’:2,3]pyrimido[1,6-a][1,3,5]triazine-3,5-dione *(**11b**)*

The compound was obtained from the reaction of **7b** with formamide in DMF as brownish crystals, which were crystallized from toluene in 90% yield, m.p. >350 °C. IR (*ν*, cm^–1^) KBr: 3062 (CH-aryl), 2944 (CH-aliph), 1686 and 1682 (2CO, amide), and 1630 (C=N). ^1^H NMR (DMSO-*d*_6_, ppm) *δ* 2.38 (s, 3 H, CH_3_), 3.99 (s, 6 H, 2OCH_3_), 4.15 (d, 1 H, *J* = 6.92 Hz, CH_2,_ thiazole), 4.22 (d, 1 H, *J* = 6.93 Hz, CH_2,_ thiazole), 5.90 (s, 1 H, thiazole), 6.72 (d, 1 H, *J* = 2.33 Hz, furan), 7.38 (s, 1H, triazine), 7.75 (d, 1 H, *J* = 2.36 Hz, furan), and 8.15 (s, 1 H, pyrimidine). ^13^C NMR (DMSO-*d*_6_) *δ* 21.8 (1C, CH_3_), 33.7 (1C, CH_2_), 62.3 (2C, 2OCH_3_), 70.15 (1C, CH, thiazole), 88.6 (1C, CH, pyrimidine), 105.4, 106.8, 116.7, 118.5, 118.9, 130.6, 138.1, 142.5, 146.3, 147.7, 148.2, 162.8, and 163.5 (13C, Ar-C), 166.9 and 170.2 (2C, 2C=O). MS (70 eV, %) *m/z* 452 (M^+^, 92%); Anal. Calc. (Found) for C_21_H_16_N_4_O_6_S (452.44): C, 55.75 (55.83); H, 3.56 (3.64); and N, 12.38 (12.44).

### 3.28. Synthesis of 8-(4-methoxy-3-methylbenzo[1,2-b:5,4- b`]difuran-2-yl)-5H,12aH-thiazolo[2’,3’:2,3] pyrimido[6,1-d][1,3,5]oxadiazepine-3,5,11(2H,10H)-trione *(**12a**)*

The compound was obtained from the reaction of **7a** with chloro-acetyl-chloride in DMF as yellow crystals, which were crystallized from dioxane in 75% yield, m.p. >350 °C. IR (*ν*, cm^–1^) KBr: 3047 (CH-aryl), 2939 (CH-aliph), 1688, 1684, and 1679 (3CO, amide), 1633 (C=N). ^1^H NMR (DMSO-*d*_6_, ppm) *δ* 2.33 (s, 3 H, CH_3_), 4.05 (s, 3 H, OCH_3_), 4.26 (d, 1 H, *J* = 6.94 Hz, CH_2,_ thiazole), 4.33 (d, 1 H, *J* = 6.95 Hz, CH_2,_ thiazole), 4.36 (d, 1 H, *J* = 6.96 Hz, CH_2,_ oxadiazepine), 4.44 (d, 1 H, *J* = 6.97 Hz, CH_2,_ oxadiazepine), 6.30 (s, 1 H, thiazole), 6.70 (d, 1 H, *J* = 2.30 Hz, furan), 7.08 (s, 1H, phenyl), 7.74 (d, 1 H, *J* = 2.31 Hz, furan), 7.90 (s, 1 H, pyrimidine). ^13^C NMR (DMSO-*d*_6_) *δ* 22.3 (1C, CH_3_), 32.5 (1C, CH_2_), 62.9 (1C, OCH_3_), 64.1 (1C, CH_2_), 68.8 (1C, CH, thiazole), 87.3 (1C, CH, pyrimidine), 102.6 (1C, CH, phenyl), 104.5, 106.4, 117.8, 118.5, 127.1, 130.7, 146.3, 152.2, 155.8, 157.2, and 158.5 (11C, Ar-C), 164.8, 167.9, and 172.9 (3C, 3C=O). MS (70 eV, %) *m/z* 453 (M^+^, 85%); Anal. Calc. (Found) for C_21_H_15_N_3_O_7_S (453.43): C, 55.63 (55.75); H, 3.33 (3.41); and N, 9.27(9.35).

### 3.29. Synthesis of 8-(4,8-dimethoxy-3-methylbenzo[1,2-b:5,4- b`]difuran-2-yl)-5H,12aH-thiazolo[2’,3’:2,3] pyrimido[6,1-d][1,3,5]oxadiazepine-3,5,11(2H,10H)-trione *(**12b**)*

The compound was obtained from the reaction of **7b** with chloro-acetyl-chloride in DMF as yellowish crystals, which were crystallized from Pet. ether in 70% yield, m.p. >350 °C. IR (*ν*, cm^–1^) KBr: 3045 (CH-aryl), 2935 (CH-aliph), 1686, 1682, and 1677 (3CO, amide), and 1631 (C=N). ^1^H NMR (DMSO-*d*_6_, ppm) *δ* 2.37 (s, 3 H, CH_3_), 4.12 (s, 6 H, 2OCH_3_), 4.28 (d, 1 H, *J* = 6.95 Hz, CH_2,_ thiazole), 4.35 (d, 1 H, *J* = 6.96 Hz, CH_2,_ thiazole), 4.38 (d, 1 H, *J* = 6.98 Hz, CH_2,_ oxadiazepine), 4.46 (d, 1 H, *J* = 6.99 Hz, CH_2,_ oxadiazepine), 6.35 (s, 1 H, thiazole), 6.73 (d, 1 H, *J* = 2.34 Hz, furan), 7.77 (d, 1 H, *J* = 2.38 Hz, furan), 7.95 (s, 1 H, pyrimidine). ^13^C NMR (DMSO-*d*_6_) *δ* 22.6 (1C,CH_3_), 32.9 (1C, CH_2_), 63.1 (2C, 2OCH_3_), 64.5 (1C, CH_2_), 69.1 (1C, CH, thiazole), 87.8 (1C, CH, pyrimidine), 105.4, 106.6, 116.5, 118.3, 118.9, 130.2, 137.9, 146.5, 147.5, 148.1, 152.2, and 158.7 (12C, Ar-C), 165.1, 168.4, and 173.2 (3C, 3C=O). MS (70 eV, %) *m/z* 483 (M^+^, 90%); Anal. Calc. (Found) for C_22_H_17_N_3_O_8_S (483.45): C, 54.66 (54.72); H, 3.54 (3.62); and N, 8.69 (8.77).

### 3.30. General Procedure for Synthesis of 2,10-di(substituted-benzylidene)-8-((4-methoxy or 4,8-dimethoxy)- 3-methylbenzo[1,2-b:5,4- b`]difuran-2-yl)-5H,12aH-thiazolo[2’,3’:2,3]pyrimido[6,1-d][1,3,5]oxadiazepine-3, 5,11 (2H,10H)-trione *(**13a–f**)*

Method A: A mix of compound **12a** (4.53 g, 0.01 mol) or **12b** (4.83 g, 0.01 mol), and the suitable aromatic aldehyde (0.02 mol) in dioxane (45 mL) containing a catalyst quantity of piperidine (0.5 mL) was stirred and refluxed for 12–15 h (TLC under control). The reaction solution was cooled, and the formed precipitate was filtered off, dried, and recrystallized from the proper solvent to give **13a**–**f**. Method B: A mixture from one of the compounds 12a (4.53 g, 0.01 mol) or 12b (4.83 g, 0.01 mol), the appropriate aromatic aldehyde (0.02 mol), and 0.02 mol of anhydrous sodium acetate was stirred under reflux in 40 mL of glacial acetic acid and 20 mL of acetic anhydride for 8–10 h. The reaction mixture was allowed to cool to room temperature and poured into cold water (100 mL). The deposited precipitate was filtered off and crystallized from an appropriate solvent to produce **13a–f**.

### 3.31. Synthesis of 2,10-di(benzylidene)-8-(4-methoxy-3-methylbenzo[1,2-b:5,4- b`]difuran-2-yl)-5H,12aH- thiazolo[2’,3’:2,3]pyrimido[6,1-d][1,3,5]oxadiazepine-3,5,11(2H,10H)-trione *(**13a**)*

The compound was obtained from the reaction of **12a** with benzaldehyde (2.12 g, 0.02 mol) in dioxane as yellowish crystals, which were crystallized from ethanol in 76% yield, m.p. >350 °C. IR (*ν*, cm^–1^) KBr: 3070 (CH-aryl), 2960 (CH-aliph), 1690, 1687, and 1684 (3CO, amide), and 1638 (C=N). ^1^H NMR (DMSO-*d*_6_, ppm) *δ* 2.28 (s, 3 H, CH_3_), 3.94 (s, 3 H, OCH_3_), 6.45 (s, 1 H, thiazole), 6.68 (d, 1 H, *J* = 2.36 Hz, furan), 7.15 (s, 1H, phenyl), 7.20–7.66 (m, 10H, phenyl), 7.72 (d, 1 H, *J* = 2.34 Hz, furan), 7.94 (s, 1H, pyrimidine), 8.04 (s, 1H, CH), and 8.10 (s, 1H, CH). ^13^C NMR (DMSO-*d*_6_) *δ* 22.5 (1C, CH_3_), 61.7 (1C, OCH_3_), 70.1 (1C, CH, thiazole), 88.4 (1C, CH, pyrimidine), 100.2 and 101.5 (2C, 2CH), 102.3 (1C, CH, phenyl), 104.1, 106.5, 117.6, 118.9, 126.8, 127.6,127.8, 128.2,128.4, 128.6, 128.7, 128.8, 128.9, 130.4, 132.1, 135.4, 139.1,146.2, 152.5, 155.6, 157.1, 158.3, and 164.5 (25C, Ar**–**C), 166.2, 168.1, and 170.4 (3C, 3C=O). MS (70 eV, %) *m/z* 629 (M^+^, 90%); Anal. Calc. (Found) for C_35_H_23_N_3_O_7_S (629.64): C, 66.77 (66.85); H, 3.68 (3.74); and N, 6.67 (6.55).

### 3.32. Synthesis of 2,10-bis(4-chlorobenzylidene)-8-(4-methoxy-3-methylbenzo[1,2-b:5,4-b`]difuran-2-yl)-5H, 12aH-thiazolo[2’,3’:2,3]pyrimido[6,1-d][1,3,5]oxadiazepine-3,5,11(2H,10H)-trione *(**13b**)*

The compound was obtained from the reaction of **12a** with 4-chlorobenzaldehyde (2.80 g, 0.02 mol) in dioxane as pale yellow crystals, which were crystallized from methanol in 80% yield, m.p. >350 °C. IR (*ν*, cm^–1^) KBr: 3074 (CH-aryl), 2963 (CH-aliph), 1689, 1686, and 1682 (3CO, amide), 1636 (C=N). ^1^H NMR (DMSO-*d*_6_, ppm) *δ* 2.29 (s, 3 H, CH_3_), 3.99 (s, 3 H, OCH_3_), 6.50 (s, 1 H, thiazole), 6.71 (d, 1 H, *J* = 2.33 Hz, furan), 7.05 (s, 1H, phenyl), 7.15–7.25 (dd, 2H, *J* = 7.40, 7.44 Hz, 4-chlorophenyl), 7.30–7.40 (dd, 2H, *J* = 7.42, 7.46 Hz, 4-chlorophenyl), 7.45–7.55 (dd, 2H, *J* = 7.46, 7.48 Hz, 4-chlorophenyl), 7.58–7.68 (dd, 2H, *J* = 7.50, 7.52 Hz, 4-chlorophenyl), 7.75 (d, 1 H, *J* = 2.37 Hz, furan), 7.90 (s, 1H, pyrimidine), 8.08 (s,1H,CH), and 8.15 (s,1H,CH). ^13^C NMR (DMSO-*d*_6_) *δ* 22.2 (1C, CH_3_), 61.3 (1C, OCH_3_), 70.4 (1C, CH, thiazole), 88.1 (1C, CH, pyrimidine), 100.4 and 101.7 (2C, 2CH), 102.5 (1C, CH, phenyl), 104.3, 106.7, 117.8, 118.5, 127.2, 128.5, 128.8, 129.3, 129.6, 130.7, 131.2, 133.1, 133.4, 133.7, 139.4, 146.5, 152.7, 155.8, 157.9, 158.5, and 164.8 (25C, Ar-C), 165.9, 168.5, and 170.8 (3C, 3C=O). MS (70 eV, %) *m/z* 698 (M^+^, 92%); Anal. Calc. (Found) for C_35_H_21_Cl_2_N_3_O_7_S (698.53): C, 60.18 (60.25); H, 3.03 (3.12); and N, 6.02 (6.10).

### 3.33. Synthesis of 8-(4-methoxy-3-methylbenzo[1,2-b:5,4- b`]difuran-2-yl)-2,10-bis(4-methoxybenzylidene)- 5H,12aH-thiazolo[2’,3’:2,3]pyrimido[6,1-d][1,3,5]oxadiazepine-3,5,11(2H,10H)-trione *(**13c**)*

The compound was obtained from the reaction of **12a** with 4-methoxybenzaldehyde (2.72 g, 0.02 mol) in dioxane as brownish crystals, which were crystallized from DMF in 78% yield, m.p. >350 °C. IR (*ν*, cm^–1^) KBr: 3072 (CH-aryl), 2966 (CH-aliph), 1687, 1684, and 1680 (3CO, amide), 1634 (C=N). ^1^H NMR (DMSO-*d*_6_, ppm) *δ* 2.30 (s, 3 H, CH_3_), 3.82 (s, 3 H, OCH_3_), 3.86 (s, 3 H, OCH_3_), 4.02 (s, 3 H, OCH_3_), 6.55 (s, 1 H, thiazole), 6.73 (d, 1 H, *J* = 2.35 Hz, furan), 7.09 (s, 1H, phenyl), 7.18–7.29 (dd, 2H, *J* = 7.60, 7.62 Hz, 4-methoxyphenyl), 7.32–7.43 (dd, 2H, *J* = 7.63, 7.65 Hz, 4-methoxyphenyl), 7.49–7.60 (dd, 2H, *J* = 7.66, 7.68 Hz, 4-methoxyphenyl), 7.62–7.73 (dd, 2H, *J* = 7.59, 7.61 Hz, 4-methoxyphenyl), 7.78 (d, 1 H, *J* = 2.33 Hz, furan), 7.96 (s, 1H, pyrimidine), 8.10 (s,1H,CH), and 8.20 (s,1H,CH). ^13^C NMR (DMSO-*d*_6_) *δ* 22.7 (1C, CH_3_), 56.2 (1C, OCH_3_), 56.8 (1C, OCH_3_), 60.6 (1C, OCH_3_), 73.5 (1C, CH, thiazole), 88.4 (1C, CH, pyrimidine), 100.6 and 101.9 (2C, 2CH), 102.8 (1C, CH, phenyl), 104.6, 106.9, 117.5, 118.7, 124.2, 124.6, 125.5, 126.9, 127.8, 129.3, 129.6, 130.4, 139.7, 144.5, 144.9, 146.2, 152.1, 155.4, 157.1, 158.3, and 164.2 (25C, Ar-C), 165.7, 168.2, and 170.1 (3C, 3C=O). MS (70 eV, %) *m/z* 689 (M^+^, 88%); Anal. Calc. (Found) for C_37_H_27_N_3_O_9_S (689.69): C, 64.44 (64.55); H, 3.95 (3.87); and N, 6.09 (6.15).

### 3.34. Synthesis of 2,10-di(benzylidene)-8-(4,8-dimethoxy-3-methylbenzo[1,2-b:5,4-b`]difuran-2-yl)-5H, 12aH -thiazolo[2’,3’:2,3]pyrimido[6,1-d][1,3,5]oxadiazepine-3,5,11(2H,10H)-trione *(**13d**)*

The compound was obtained from the reaction of **12b** with benzaldehyde (2.12 g, 0.02 mol) in dioxane as yellow crystals, which were crystallized from benzene in 74% yield, m.p. >350 °C. IR (*ν*, cm^–1^) KBr: 3075 (CH-aryl), 2964 (CH-aliph), 1689, 1686, and 1683 (3CO, amide), 1637 (C=N). ^1^H NMR (DMSO-*d*_6_, ppm) *δ* 2.27 (s, 3 H, CH_3_), 3.98 (s, 6 H, 2OCH_3_), 6.48 (s, 1 H, thiazole), 6.66 (d, 1 H, *J* = 2.31 Hz, furan), 7.14–7.62 (m, 10H, phenyl), 7.68 (d, 1 H, *J* = 2.32 Hz, furan), 7.91 (s, 1H, pyrimidine), 8.03 (s, 1H, CH), and 8.07 (s, 1H, CH). ^13^C NMR (DMSO-*d*_6_) *δ* 22.6 (1C, CH_3_), 62.8 (2C, 2OCH_3_), 71.2 (1C, CH, thiazole), 88.7 (1C, CH, pyrimidine), 100.4 and 101.8 (2C, 2CH), 105.5, 106.8, 116.5, 118.2, 118.8, 127.7, 127.9, 128.1, 128.3, 128.5, 128.6, 128.7, 128.8, 130.1, 132.5, 135.6, 138.2, 139.3, 146.5, 147.3, 147.8, 152.4, 158.6, and 164.7 (26C, Ar-C), 165.8, 167.7, and 169.9 (3C, 3C=O). MS (70 eV, %) *m/z* 659 (M^+^, 88%); Anal. Calc. (Found) for C_36_H_25_N_3_O_8_S (659.67): C, 65.55 (65.64); H, 3.82 (3.76); and N, 6.37 (6.44).

### 3.35. Synthesis of 2,10-bis(4-chlorobenzylidene)-8-(4,8-dimethoxy-3-methylbenzo[1,2-b:5,4-b`]difuran-2-yl)- 5H,12aH-thiazolo[2’,3’:2,3]pyrimido[6,1-d][1,3,5]oxadiazepine-3,5,11(2H,10H)-trione *(**13e**)*

The compound was obtained from the reaction of **12b** with 4-chlorobenzaldehyde (2.80 g, 0.02 mol) in dioxane as yellowish crystals, which were crystallized from acetone in 73% yield, m.p. >350 °C. IR (*ν*, cm^–1^) KBr: 3072 (CH-aryl), 2961 (CH-aliph), 1687, 1684, and 1680 (3CO, amide), 1633 (C=N). ^1^H NMR (DMSO-*d*_6_, ppm) *δ* 2.32 (s, 3 H, CH_3_), 4.10 (s, 6 H, 2OCH_3_), 6.55 (s, 1 H, thiazole), 6.67 (d, 1 H, *J* = 2.30 Hz, furan), 7.09–7.20 (dd, 2H, *J* = 7.48, 7.50 Hz, 4-chlorophenyl), 7.25–7.36 (dd, 2H, *J* = 7.52, 7.54 Hz, 4-chlorophenyl), 7.40–7.51 (dd, 2H, *J* = 7.55, 7.57 Hz, 4-chlorophenyl), 7.55–7.66 (dd, 2H, *J* = 7.56, 7.59 Hz, 4-chlorophenyl), 7.71 (d, 1 H, *J* = 2.32 Hz, furan), 7.85 (s, 1H, pyrimidine), 8.04 (s, 1H, CH), 8.11 (s, 1H, CH). ^13^C NMR (DMSO-*d*_6_) *δ* 21.8 (1C, CH_3_), 61.7 (2C, 2OCH_3_), 70.6 (1C, CH, thiazole), 88.6 (1C, CH, pyrimidine), 100.8 and 101.9 (2C, 2CH), 105.6, 106.3, 116.7, 118.5, 118.8, 128.5, 128.9, 129.2, 129.6, 130.3, 130.9, 133.2, 133.6, 133.9, 138.1, 139.7, 146.2, 147.4, 148.1, 152.3, 158.4, and 164.9 (26C, Ar-C), 166.1, 168.8, and 171.2 (3C, 3C=O). MS (70 eV, %) *m/z* 728 (M^+^, 95%); Anal. Calc. (Found) for C_36_H_23_Cl_2_N_3_O_8_S (728.55): C, 59.35 (59.43); H, 3.18 (3.24); and N, 5.77 (5.85).

### 3.36. Synthesis of 8-(4,8-dimethoxy-3-methylbenzo[1,2-b:5,4-b`]difuran-2-yl)-2,10-bis(4-methoxybenzylidene)-5H,12aH-thiazolo[2’,3’:2,3]pyrimido[6,1-d][1,3,5]oxadiazepine-3,5,11(2H,10H)-trione *(**13f**)*

The compound was obtained from the reaction of **12b** with 4-methoxybenzaldehyde (2.72 g, 0.02 mol) in dioxane as brown crystals, which were crystallized from hexane in 70% yield, m.p. >350 °C. IR (*ν*, cm^–1^) KBr: 3068 (CH-aryl), 2961 (CH-aliph), 1685, 1681, and 1678 (3CO, amide), 1629 (C=N). ^1^H NMR (DMSO-*d*_6_, ppm) *δ* 2.35 (s, 3 H, CH_3_), 3.84 (s, 3H, OCH_3_), 3.92 (s, 3H, OCH_3_), 4.04 (s, 6 H, 2OCH_3_), 6.58 (s, 1 H, thiazole), 6.69 (d, 1 H, *J* = 2.34 Hz, furan), 7.13–7.25 (dd, 2H, *J* = 7.61, 7.64 Hz, 4-methoxyphenyl), 7.28–7.40 (dd, 2H, *J* = 7.65, 7.67 Hz, 4-methoxyphenyl), 7.44–7.55 (dd, 2H, *J* = 7.63, 7.68 Hz, 4-methoxyphenyl), 7.58–7.70 (dd, 2H, *J* = 7.62, 7.66 Hz, 4-methoxy phenyl), 7.70 (d, 1H, *J* = 2.37 Hz, furan), 7.90 (s, 1H, pyrimidine), 8.15 (s, 1H, CH), and 8.24 (s, 1H, CH). ^13^C NMR (DMSO-*d*_6_) *δ* 22.5 (1C, CH_3_), 56.5 (1C, OCH_3_), 56.9 (1C, OCH_3_), 61.2 (2C, 2OCH_3_), 73.1 (1C, CH, thiazole), 88.7 (1C, CH, pyrimidine), 100.3 and 101.6 (2C, 2CH), 105.4, 106.5, 116.8, 118.5, 118.9, 124.4, 124.8, 125.1, 127.2, 129.6, 129.8, 130.5, 135.3, 135.7, 138.1, 139.3, 146.2, 147.4, 147.8, 152.3, 157.6, and 164.7 (26C, Ar-C), 165.7, 168.2, and 170.1 (3C, 3C=O). MS (70 eV, %) *m/z* 719 (M^+^, 94%); Anal. Calc. (Found) for C_38_H_29_N_3_O_10_S (719.72): C, 63.42 (63.51); H, 4.06 (4.12); and N, 5.84 (5.75).

### 3.37. Biological Screening

#### 3.37.1. Ethics Approval and Consent to Participate

All the procedures adopted were approved and agreed upon by Animal Ethical committee of Mansoura University, Faculty of Pharmacy (Department of Pharmacognosy), 35516, Egypt.

#### 3.37.2. Human and Animal Rights

No humans were used in the study. The research was conducted in accordance with the ethical standards. All care and use guidelines for laboratory animals were followed.

#### 3.37.3. Chemicals and Drugs

Carrageenan and glacial acetic acid were purchased from Sigma-Aldrich (St. Louis, Mo, USA). Celecoxib, indomethacin, and diclofenac sodium were purchased from Pfizer (New York, NY, USA). The interleukin 1 beta (IL-1 b) ELISA Kit was purchased from Reddot Biotech. Inc. (Kelowna, BC, Canada).

#### 3.37.4. Animals

Male albino rats and mice were used to evaluate anti-inflammatory and analgesic activities, respectively. Animals were purchased from the animal house at The Holding Company for biological products, vaccines, and drugs (VACSERA, Giza, Egypt), and they were acclimatized for two weeks before the experiments at the animal house of Mansoura University, Faculty of Pharmacy (Department of Pharmacognosy). The animals were divided into different groups, five animals in each group. Food and water were supplied ad libitum to the animals.

#### 3.37.5. In Vitro COX Assay

All new compounds **4**–**13** and derivatives were screened for in vitro COX inhibitory activity (Ibrahim et al. [37]) using an enzyme immunoassay (EIA) kit (Cayman Chemical Company, USA). The IC50 values for the compounds were determined using celecoxib as the reference drug, which is a selective COX-2 inhibitor medicine. Furthermore, the selectivity index for COX-2 (COX-1 IC_50_ / COX-2 IC_50_) was calculated for the compounds. The ability of the compounds to inhibit ovine COX-1 and the human recombinant COX-2 form was determined and evaluated using a COX inhibitor Screening Assay kit (catalogue no. 560131, Cayman Chemical, Ann Arbor, MI, USA) in agreement with instructions recommended and mentioned by the supplier.

#### 3.37.6. Screening of Analgesic Activity

The analgesic activity of the compounds was performed using the writhing test as described by Gawade et al. [35]. Mice weight ranged from 25 to 30 g. Then, mice were divided into 18 different groups, each containing five animals. The animals fasted for 8 h before the experiment. Administration of the compounds (10 mg/kg, p.o.) was performed orally. Equal doses of diclofenac as the reference drug and saline as a negative control were also administrated orally. Writhing was induced one hour after compound administration by using 0.1 mL of 1% glacial acetic acid at a volume of 0.1 mL/10 g body weight. The number of writhing responses (stretching of the abdomen, extension of hind limbs, twisting of the trunk, and elongation of the body) observed was counted for 20 min. The protection percentile against acetic acid-induced writhing was calculated according to the following formula:

(1) $$\mathrm{Analgesic}\;\mathrm{activity}\;\left(\%\right)\;=\frac{n-n\prime }{n}\times 100*$$

where *n* is the mean number of writhes of the control group, and *n*’ is the mean number of writhes of the tested compound group.

#### 3.37.7. Evaluation of the Anti-Inflammatory Activity

The method of Paw edema reported by Puttaswamy et al. [36] was used to evaluate and screen the activity of the compounds that exhibited analgesic activity against inflammation. Albino rats (150–180 g) were used. The rats were divided into 11 experimental groups of five rats each. One hour after oral administration of the compounds (10 mg/kg), 0.1 mL of 1% carrageenan solution was injected in the left hind paw of each animal. Rat paw volumes were measured with a digital caliber plethysmometer (UGO Basile, Varese, Italy) used to measure inflammation height at 0, 1, 2, and 3 h after carrageenan injection. The percentage increase in inflammation in the left hind paw, in comparison to the uninjected right hind paw, was determined, calculated, and expressed as the amount of inflammation. All the tested compounds were orally administered at equivalent doses of the reference drug diclofenac (10 mg/kg, p. o.). The anti-inflammatory activity of the selected analgesic compounds after 3 h was expressed as percentage inhibition of edema, which was calculated according to the following equation:

(2) $$\mathrm{Inhibition}\;\mathrm{of}\;\mathrm{edema}\;\left(\%\right)\;=\frac{Vcont-Vtest}{Vcont}\times 100*$$

where *V cont* is the edema volume in the control group, and *V test* is the edema volume in the group of the screened compound.

#### 3.37.8. ELISA Determination of IL1

IL1 is one of the important inflammatory mediators and is used to confirm the anti-inflammatory activities of new anti-inflammatory compounds. At the end of the paw edema test, blood samples were collected from the orbital axis of the eye under light ether anesthesia. Sera were separated after centrifugation of blood at 5000 rpm for 15 min; the sera were stored at –80 ^o^ C until the ELISA assay was performed according to the manufacturer instructions.

#### 3.37.9. Statistical Analysis

Data were expressed as mean ± SEM. One-way analysis of variance, (ANOVA, GraphPad Software, La Jolla California, USA) followed by Bonferroni’s post hoc test was used to analyze the data using the Statistical Package for Social Sciences, version 19 (SPSS, Inc., Chicago, IL, USA). *P* < 0.05 was considered to be statistically significant.

## 4. Conclusions

The new heterocyclic compounds were evidenced to be interesting for the study of anti-inflammatory and analgesic activities, which afforded the data of COX-1 and COX-2 inhibition as shown in Table 1, Table 2, Table 3 and Table 4. Thus, this research offers rapid, effective, and new procedures for the synthesis of new heterocyclic compounds in high yields, including 2-((benzofuran-6-yl) oxy)-*N*- (6-oxo-2-thioxopyrimidinyl) acetamide **(4a**–**b)**, 3-methyl-*N*-(2-thioxopyrimidinyl)benzo[1,2-b: 5,4-b`]difuran-2-carboxamide **(5a**–**b),** 2-((4-(3-methylbenzo[1,2-b:5,4-b`]difuran-2-carboxamido)-oxo- pyrimidin-2-yl)thio) acetic acid **(6a**–**b)**, *N*-(3,5-dioxothiazolo[3,2-a]pyrimidinyl)-3-methylbenzo[1,2- b:5,4-b`]difuran-2-carboxamide **(7a**–**b)**, *N*-(2-thioxopyrimidinyl)-3-methylbenzo[1,2-b:5,4-b`]difuran -2-carbimidoylchloride **(8a**–**b)**, *N*-(6-chloro-2-(methylthio) pyrimidinyl)-3-methylbenzo[1–b:5,4-b`] difuran-2-carbimidoyl **(9a**–**b)**, *N*-(2,6-di(piperazinyl or morpholino) pyrimidinyl)-1-(3-methylbenzo [1,2-b:5,4-b`]difuranyl)-1-(piperazinyl or morpholino) methanimine **(10a**–**d)**, 8-(3-methylbenzo[1,2-b: 5,4-b`]difuranyl)-thiazolo[2’,3’:2,3]pyrimido[1,6-a][1,3,5]triazine-3,5-dione (**11a**–**b)**, 8-(3-methylbenzo [1–-b: 5,4-b`]difuranyl)-thiazolo[2’,3’:2,3]pyrimido[6,1-d][1,3,5]oxadiazepine-3,5,11-trione **(12a**–**b)**, and 2,10-di (sub-benzylidene)-8-(3-methylbenzo[1,2-b:5,4-b`]difuranyl)- thiazolo[2’,3’:2,3]pyrimido [6,1-d][1,3,5]oxadiazepine-3, 5, 11-trione **(13a**–**f).** The compounds **10a**–**d**, **13a**–**f,** and **12a**–**b** exhibited the highest anti-inflammatory and analgesic activities (COX-1/COX-2), because these compounds contain various functional groups such as piperazine, morpholine, triazine, oxadiazepine, thiazole, pyrimidine, furan, 4-chlorophenyl, 4-methoxyphenyl ring, methoxy, methyl, and amino groups.

## Figures and Tables

**Table 1:** IC_50_ and selectivity index values of the compounds.

Compound	COX1_IC_50_ μM ^a^	COX2_IC_50_ μM ^a^	COX2_(SI) ^b^
**Celecoxib**	**15.80**	**0.057**	**277**
**Diclofenac**	**4.7**	**0.920**	**5.1**
**4a**	18.80	0.460	41
**4b**	18.50	0.411	45
**5a**	18.10	0.375	48
**5b**	17.88	0.360	50
**6a**	17.50	0.345	51
**6b**	17.10	0.311	55
**7a**	7.95	0.101	79
**7b**	7.18	0.090	80
**8a**	16.95	0.280	61
**8b**	15.50	0.245	63
**9a**	11.95	0.170	70
**9b**	11.10	0.155	72
**10a**	4.23	0.043	98
**10b**	4.60	0.048	96
**10c**	4.15	0.042	99
**10d**	4.45	0.046	97
**11a**	6.85	0.085	81
**11b**	6.60	0.080	83
**12a**	6.40	0.075	85
**12b**	6.10	0.070	87
**13a**	5.79	0.064	90
**13b**	5.20	0.055	95
**13c**	5.52	0.060	92
**13d**	5.71	0.063	91
**13e**	5.10	0.053	96
**13f**	5.45	0.058	94

(**a**) The concentration of the compound producing 50% inhibition of ovine cyclooxygenase-1 (COX-1) and cyclooxygenase-2 (COX-2) enzymes. The result is the mean of two determinations obtained by assays of enzyme kits (Cayman Chemicals Inc., Ann Arbor, MI, USA). (**b**) The in vitro COX-2 selectivity index (COX-1 IC50 / COX-2 IC_50_).

**Table 2:** Analgesic activity of the compounds using the writhing test.

Compound	Writhing Number in 20 min (Mean ± SEM)	Analgesic Activity (% protection)
**Control**	**40 ± 0.5**	**0**
**Diclofenac**	**25 ± 1.2 ^a^**	**51 ± 1.15 ^a^**
**4a**	58 ± 1.5 ^a,b^	18 ± 1.20
**4b**	56 ± 1.2 ^a,b^	19 ± 1.30
**5a**	54 ± 1.6 ^a,b^	21 ± 2.10
**5b**	52 ± 1.4 ^a,b^	23 ± 0.70
**6a**	50 ± 1.6 ^a,b^	25 ± 0.50
**6b**	49 ± 1.2 ^a,b^	26 ± 0.80
**7a**	42 ± 1.8 ^a,b^	33 ± 1.70
**7b**	40 ± 1.5 ^a,b^	35 ± 1.14
**8a**	48 ± 1.9 ^a,b^	27 ± 0.60
**8b**	47 ± 2.2 ^a,b^	28 ± 0.95
**9a**	45 ± 2.4 ^a,b^	30 ± 1.05
**9b**	44 ± 2.5 ^a,b^	32 ± 1.2
**10a**	25 ± 1.3 ^a^	50 ± 1.6 ^a^
**10b**	27 ± 1.1 ^a^	48 ± 1.5 ^a^
**10c**	24 ± 1.5 ^a^	51 ± 1.4 ^a^
**10d**	26 ± 1.2 ^a^	49 ± 1.1 ^a^
**11a**	39 ± 2.1 ^a^	36 ± 0.75 ^a^
**11b**	37 ± 2.3 ^a^	38 ± 0.80 ^a^
**12a**	35 ± 1.2 ^a^	40 ± 1.50 ^a^
**12b**	34 ± 1.7 ^a^	41 ± 1.10 ^a^
**13a**	33 ± 1.9 ^a^	42 ± 1.90 ^a^
**13b**	29 ± 1.5 ^a^	46 ± 1.6 ^a^
**13c**	31 ± 1.1 ^a^	44 ± 0.85 ^a^
**13d**	32 ± 1.4 ^a^	43 ± 1.90 ^a^
**13e**	28 ± 1.3 ^a^	47 ± 1.7 ^a^
**13f**	30 ± 1.8 ^a^	45 ± 0.95 ^a^

Values are expressed as mean ± SEM (*n* = 5). Data were analyzed using one-way ANOVA followed by a Bonferroni post hoc test. A *p-*value was considered significant if it was less than 0.05 compared to control; (**a**) Significantly different from the control group; (**b**) Significantly different from the diclofenac group.

**Table 3:** Percentage change and inhibition in rat paw height after carrageenan-Induced paw edema in rats.

Compound	% Change in Paw Height (Mean ± SEM)			Anti-Inflammatory Activity (% Inhibition of Edema)
	1 hr	2 hr	3 hr	3 hr
**Control**	**0.36 ± 0.01**	**0.44 ± 0.01**	**0.50 ± 0.01**	**---------**
**Diclofenac**	**0.15 ± 0.03 ^a^**	**0.13 ± 0.02 ^a^**	**0.20 ± 0.01 ^a^**	**63**
**4a**	0.49 ± 0.01 ^a^	0.40 ± 0.02 ^a^	0.49 ± 0.02 ^a^	24
**4b**	0.47 ± 0.04 ^a^	0.38 ± 0.03 ^a^	0.47 ± 0.01 ^a^	26
**5a**	0.45 ± 0.03 ^a^	0.36 ± 0.02 ^a^	0.46 ± 0.02 ^a^	28
**5b**	0.42 ± 0.04 ^a^	0.34 ± 0.01 ^a^	0.44 ± 0.03 ^a^	30
**6a**	0.39 ± 0.01 ^a^	0.32 ± 0.02 ^a^	0.41 ± 0.02 ^a^	35
**6b**	0.38 ± 0.03 ^a^	0.31 ± 0.04 ^a^	0.39 ± 0.01 ^a^	38
**7a**	0.31 ± 0.01 ^a^	0.26 ± 0.02 ^a^	0.30 ± 0.02 ^a^	50
**7b**	0.30 ± 0.02 ^a^	0.25 ± 0.01 ^a^	0.29 ± 0.01 ^a^	52
**8a**	0.37 ± 0.02 ^a^	0.30 ± 0.03 ^a^	0.37 ± 0.02 ^a^	40
**8b**	0.36 ± 0.01 ^a^	0.29 ± 0.02 ^a^	0.35 ± 0.02 ^a^	42
**9a**	0.35 ± 0.01 ^a^	0.28 ± 0.02 ^a^	0.33 ± 0.02 ^a^	45
**9b**	0.33 ± 0.02 ^a^	0.27 ± 0.01 ^a^	0.31 ± 0.01 ^a^	48
**10a**	0.19 ± 0.01 ^a^	0.14 ± 0.02 ^a^	0.16 ± 0.02 ^a^	67
**10b**	0.21 ± 0.02 ^a^	0.16 ± 0.01 ^a^	0.18 ± 0.01 ^a^	65
**10c**	0.18 ± 0.01 ^a^	0.13 ± 0.02 ^a^	0.15 ± 0.02 ^a^	68
**10d**	0.20 ± 0.01 ^a^	0.15 ± 0.02 ^a^	0.17 ± 0.02 ^a^	66
**11a**	0.30 ± 0.02 ^a^	0.24 ± 0.01 ^a^	0.28 ± 0.01 ^a^	55
**11b**	0.29 ± 0.01 ^a^	0.23 ± 0.01 ^a^	0.27 ± 0.02 ^a^	56
**12a**	0.28 ± 0.02 ^a^	0.23 ± 0.01 ^a^	0.26 ± 0.01 ^a^	57
**12b**	0.28 ± 0.01 ^a^	0.22 ± 0.02 ^a^	0.25 ± 0.02 ^a^	58
**13a**	0.27 ± 0.02 ^a^	0.21 ± 0.01 ^a^	0.24 ± 0.01 ^a^	59
**13b**	0.23 ± 0.02 ^a^	0.18 ± 0.01 ^a^	0.20 ± 0.02 ^a^	63
**13c**	0.25 ± 0.01 ^a^	0.19 ± 0.02 ^a^	0.22 ± 0.02 ^a^	61
**13d**	0.26 ± 0.01 ^a^	0.20 ± 0.02 ^a^	0.23 ± 0.02 ^a^	60
**13e**	0.22 ± 0.01 ^a^	0.17 ± 0.02 ^a^	0.19 ± 0.02 ^a^	64
**13f**	0.24 ± 0.02 ^a^	0.18 ± 0.02 ^a^	0.21 ± 0.01 ^a^	62

Values are expressed as mean ± SEM (*n* = 5). Data were analyzed using one-way ANOVA followed by a Bonferroni post hoc test. A *p*-value was considered significant if it was less than 0.05 compared to control; **^a^**Significantly different from the control group; **^b^** Significantly different from the diclofenac group.

**Table 4:** Serum level of interleukin-1 beta (IL-1 β) after paw edema in rats.

Groups	IL-1 β Concentration (Pg /mL)
Normal animals	20 ± 1.82
Paw edema-bearing animals	44 ± 0.75 ^a^
10a	31 ± 0.55 ^b^
10b	33 ± 0.38 ^b^
10c	30 ± 0.70 ^b^
10d	32 ± 0.45 ^b^
11a	43 ± 1.15 ^b^
11b	42 ± 0.98 ^b^
12a	41 ± 0.85 ^b^
12b	40 ± 0.22 ^b^
13a	39 ± 0.31 ^b^
13b	35 ± 0.28 ^b^
13c	37 ± 0.57 ^b^
13d	38 ± 0.62 ^b^
13e	34 ± 0.33 ^b^
13f	36 ± 0.25 ^b^

Values are expressed as mean ± SEM (*n* = 5). Data were analyzed using one-way ANOVA followed by the Bonferroni test as a post hoc test. A *p*-value was considered as significant if it was less than 0.05 compared to control; **^a^**Significant from normal animals; **^b^** Significant from paw edema-bearing animals.
